# DIS3L2 knockdown impairs key oncogenic properties of colorectal cancer cells via the mTOR signaling pathway

**DOI:** 10.1007/s00018-023-04833-5

**Published:** 2023-06-20

**Authors:** Juan F. García-Moreno, Rafaela Lacerda, Paulo J. da Costa, Marcelo Pereira, Margarida Gama-Carvalho, Paulo Matos, Luísa Romão

**Affiliations:** 1 Departamento de Genética Humana, Instituto Nacional de Saúde Doutor Ricardo Jorge, 1649-016 Lisbon, Portugal; 2 Faculdade de Ciências, BioISI – Instituto de Biossistemas e Ciências Integrativas, Universidade de Lisboa, 1749-016 Lisbon, Portugal

**Keywords:** DIS3L2, Colorectal cancer, Cell viability, mTOR, AZGP1, Cell migration

## Abstract

DIS3L2 degrades different types of RNAs in an exosome-independent manner including mRNAs and several types of non-coding RNAs. DIS3L2-mediated degradation is preceded by the addition of nontemplated uridines at the 3’end of its targets by the terminal uridylyl transferases 4 and 7. Most of the literature that concerns DIS3L2 characterizes its involvement in several RNA degradation pathways, however, there is some evidence that its dysregulated activity may contribute to cancer development. In the present study, we characterize the role of DIS3L2 in human colorectal cancer (CRC). Using the public RNA datasets from The Cancer Genome Atlas (TCGA), we found higher DIS3L2 mRNA levels in CRC tissues versus normal colonic samples as well as worse prognosis in patients with high DIS3L2 expression. In addition, our RNA deep-sequencing data revealed that knockdown (KD) of DIS3L2 induces a strong transcriptomic disturbance in SW480 CRC cells. Moreover, gene ontology (GO) analysis of significant upregulated transcripts displays enrichment in mRNAs encoding proteins involved in cell cycle regulation and cancer-related pathways, which guided us to evaluate which specific hallmarks of cancer are differentially regulated by DIS3L2. To do so, we employed four CRC cell lines (HCT116, SW480, Caco-2 and HT-29) differing in their mutational background and oncogenicity. We demonstrate that depletion of DIS3L2 results in reduced cell viability of highly oncogenic SW480 and HCT116 CRC cells, but had little or no impact in the more differentiated Caco-2 and HT-29 cells. Remarkably, the mTOR signaling pathway, crucial for cell survival and growth, is downregulated after DIS3L2 KD, whereas AZGP1, an mTOR pathway inhibitor, is upregulated. Furthermore, our results indicate that depletion of DIS3L2 disturbs metastasis-associated properties, such as cell migration and invasion, only in highly oncogenic CRC cells. Our work reveals for the first time a role for DIS3L2 in sustaining CRC cell proliferation and provides evidence that this ribonuclease is required to support the viability and invasive behavior of dedifferentiated CRC cells.

**Supplementary Information:**

The online version contains supplementary material available at 10.1007/s00018-023-04833-5.

## Introduction

DIS3-like 3′–5′ exoribonuclease 2, DIS3L2, a member of the highly conserved RNase II/RNB family, attracted much research interest when, in 2012, Astuti and colleagues identified germline mutations in the *DIS3L2* gene in patients affected by the Perlman syndrome, a congenital overgrowth condition associated with Wilms tumor susceptibility [1]. In the same study, the authors also reported the first insights into DIS3L2 cytoplasmic exoribonuclease activity in human embryonic HEK293 cells. DIS3L2 is composed of two cold-shock domains (CDS domains) at the N-terminus followed by a ribonuclease II domain (RNB domain) responsible for its catalytic activity, and a carboxy-terminal S1 domain. Unlike DIS3L2, its two other human paralogs, DIS3 and DIS3L1, contain a PilT N-terminus (PIN) domain that allows them to operate as the catalytic subunits of the exosome complex [2–4]. The lack of this domain, makes DIS3L2 the only major eukaryotic 3′–5′ exoribonuclease capable of degrading its targets in an exosome-independent manner [5, 6]. In the cytoplasm, DIS3L2 is involved in an uridylation-dependent mRNA degradation. Biochemical and structural approaches revealed that oligo-uridylation at the 3’-end, largely performed by the terminal uridylyl transferases 4 and 7 (TUTs), serves as a recognition signal for DIS3L2 to bind and degrade its targets [5, 7–9]. The DIS3L2/uridylation pathway was described for the first time in *Schizosaccharomyces pombe*, in which DIS3L2 was observed to execute its nuclease activity over uridylated poly(A)-containing mRNAs [5]. In mammalian cells, the let-7 miRNA precursor (pre-let-7) was identified as the first uridylated target of DIS3L2, which suppresses let-7 miRNA biogenesis [8, 10].

Several biological and physiological processes are significantly affected by the action of DIS3L2, including RNA quality control, as seen for the nonsense-mediated mRNA decay (NMD) pathway [7, 9], cell proliferation and tissue growth [11, 12], cell division [1], cell differentiation [8, 10] and apoptosis [13]. Therefore, an abnormal activity of DIS3L2 is expected to participate in the etiology of several human disorders, as seen for the first time in the Perlman syndrome [1]. Some studies have associated DIS3L2 with tumorigenesis and cancer-related processes, however, its role in cancer development and progression is still unclear. Consistently with its linkage within the Perlman syndrome families, Astuti et al. also detected *DIS3L2* mutations in sporadic Wilms tumors [1]. Moreover, they showed in HeLa cervical cancer cells that loss of *DIS3L2* expression is associated with multiple chromosomal abnormalities, mitotic errors as well as cell death phenotypes. These findings prompted further research of the role of DIS3L2 in cell proliferation and its involvement in the cell cycle. Towler and colleagues found out that DIS3L2 is required for a proper control of proliferation in the wing imaginal discs of *Drosophila melanogaster*, since its knockdown (KD) results in wing overgrowth due to higher cell proliferation [12]. In a more recent work, using DIS3L2 null mutants in *Drosophila*, these authors identified a conserved growth factor called Imaginal Disc Growth Factor 2 (Idfg2) as the DIS3L2 target responsible for causing the overgrowth phenotype [14]. RNA-seq analysis over DIS3L2-null nephron progenitor cells from mouse, which serves as model for Wilms tumor research, revealed another DIS3L2 target, the insulin growth factor 2 (IGF2) [15] that has been reported as an oncogene driving Wilms tumorigenesis [16]. These findings suggest a tumor suppressor role for DIS3L2 in this rare kidney cancer.

However, there are several reports of tumor promoting roles for DIS3L2. Many of these describe its cooperation with LIN28 to suppress maturation of pre-miRNA let-7 in several human cell lines and mouse embryonic cells [8, 10, 17, 18]. More specifically, the RNA-binding protein LIN28 binds pre-miRNA let-7 and recruits TUT4 and TUT7, promoting uridylation, which allows DIS3L2 to recognize and degrade pre-let-7. LIN28 is a pluripotency factor crucial to maintain an undifferentiated and proliferative state in stem cells during development by blocking let-7 expression [19, 20], and functions as oncoprotein in a wide variety of human cancers [21, 22]. On the other hand, let-7 miRNA is a key regulator of embryonic cell differentiation and a tumor suppressor that affects various types of cancer [23–25]. In addition, clustering analysis of expression profiles has shown that LIN28-positive cancer cells also display high expression of DIS3L2 and uridylation factors as well as miRNA let-7 targets [18]. These results together with the fact that let-7 is a tumor suppressor, suggest that by inhibiting let-7 biogenesis, DIS3L2 can, consequently, promote tumorigenesis. Another important study documented a pro-tumorigenic role for DIS3L2 in human hepatocellular carcinoma, via regulation of alternative splicing [26]. DIS3L2 interacts with the heterogeneous nuclear ribonucleoprotein (hnRNP) U through its cold-shock domains and regulates the alternative splicing of the small GTPase RAC1’s pre-mRNA, yielding the production of the tumor-promoting splicing variant, RAC1B. Altogether, these findings indicate that DIS3L2 could behave as tumor suppressor or tumor promotor depending on the cancer type.

In this work, we unveil that proper DIS3L2 expression is crucial for colorectal cancer (CRC) cells to maintain key tumorigenic properties. Our initial observations indicate that DIS3L2 is overexpressed in CRC tissues versus non-tumorigenic samples, according to The Cancer Genome Atlas (TCGA) database. In addition, depletion of DIS3L2 reduces the viability of highly oncogenic SW480 and HCT116 CRC cells. We also found that the mTOR signaling pathway, crucial for cell viability and survival, is suppressed upon DIS3L2 KD in those cell lines. Moreover, loss of DIS3L2 expression impairs SW480 and HCT116 cell migration and invasion. Interestingly, interference with DIS3L2 expression does not significantly affect any of these properties in the low oncogenic Caco-2 and HT-29 CRC cell lines, nor in control NCM460 non-transformed colonocytes. Collectively, these results contribute to improve our understanding of DIS3L2 function in key hallmarks of cancer and suggest a pro-tumorigenic role for this ribonuclease in advanced CRCs.

## Materials and methods

### Clinical data and samples

Retrieval of cancer genomic datasets and associated clinical data was accomplished by using the UCSCXenaTool R package (v.1.4.8) [27]. RNA-seq gene expression data (IlluminaHiSeq RNASeqV2) from tumor tissues (“primary tumor”, “recurrent tumor” and “metastatic”) and matched normal samples (“solid tissue normal”) were downloaded from The Cancer Genome Atlas (TCGA). The TCGA database has a total of 736 colorectal cancer patients. Gene expression profiles for DIS3L2 were available in a total of 434 patients. For survival analysis, CRC patients were divided into two groups: high and low expression groups, based on the FPKM value of DIS3L2. The prognosis of each subject is calculated by Kaplan–Meier estimators and comparison between groups is performed with log-rank tests [28]. The expression cut-off value was selected based on the FPKM value that yields maximal difference with regard to overall survival between the two groups at the lowest log-rank P value. Therefore, the optimal cut-off is defined as the point with the most significant split. The Survminer (v.0.4.9) and Survival (3.3.1) R packages were applied to generate Kaplan–Meier survival plots.

### Cell lines and cell cultures

The non-transformed colonic epithelial cell line NCM460 and CRC cell lines (SW480, HCT116, Caco-2 and HT-29) were cultured in the following media: NCM460, Caco-2 and HT-29 cells were grown in Roswell Park Memorial Institute 1640 (RPMI; Gibco, Thermo Fisher Scientific, Waltham, MA, USA) supplemented with 10% (v/v) fetal bovine serum (FBS; Gibco, Thermo Fisher Scientific). SW480 cells were cultured in Dulbecco’s Modified Eagle Medium (DMEM; Gibco, Thermo Fisher Scientific) supplemented with 10% (v/v) FBS. HCT116 cells were grown in McCoy’s 5A (Modified) Medium supplemented with 10% (v/v) FBS. Cells were maintained in an incubator at 37 °C, in a humidified atmosphere with 5% (v/v) CO_2_.

### Cell transfections and mRNA half-life analysis

For gene silencing, cells were seeded at 30–40% confluence and 24 h later, siRNAs targeting DIS3L2 (5′-GCACCAAACUUAGCUACGA-3′), TUT4 (5′-GGAUUUGGAUUUCGUGAUA-3′), TUT7 (5′-AUACGUUUAAUUUCCAGCC-3′), or Luciferase (LUC; 5′- CGUACGCGGAAUACUUCGA-3′), the latter used as a control condition (Thermo Fisher Scientific), were transiently transfected using Lipofectamine 2000 Transfection Reagent (Invitrogen, Waltham, MA, USA) following the manufacturer’s instructions. For overexpression studies, cells were seeded at 70–80% confluence and 24 h after, were transiently transfected using Lipofectamine 2000 (as above) with the following plasmids, as indicated: p3XFLAG-CMV^™^-10 expression vector encoding the human wild type version of DIS3L2; pCK-FLAG-TUT4 and pCK-FLAG-TUT7 vectors, and the empty commercial vector pcDNA3.1 + (Invitrogen, Waltham, MA, USA), which was used as control. For both, silencing and overexpression approaches, cells were harvested 24, 48 or 72 h later, depending on the experimental objective. The size of cell culture plates and the amount of each reagent are specified in each experimental method along this section.

For mRNA half-life analysis, SW480 cells were seeded at 30–40% confluence in 35-mm well plates and 24 h later, 250 pmol of siRNAs targeting DIS3L2 or LUC were transiently transfected as described above. Forty-eight hours after siRNA transfection, cells were treated with 60 µM of adenosine analogue, 5,6-Dichloro-1-β-D-Ribo-furanosyl-benzimidazole (DRB; D1916, Sigma-Aldrich) to inhibit transcription, and were then harvested at different time points (0, 1, 2, and 4 h).

### RNA extraction and cDNA synthesis

Total RNA extracts were initially obtained by lysis of transiently transfected cells. Briefly, cells were washed with 1 mL of pre-chilled 1× (v/v) phosphate-buffered saline (PBS 1×) and lysed in 100 µL of NP40 buffer [50 mM Tris–HCl pH = 7.5, 10 mM MgCl_2_, 100 mM NaCl, 10% (v/v) glycerol and 1% (v/v) Nonidet P-40 (Roche)] containing RNase inhibitor (NZYTech, Portugal). Then, lysis solution was collected, and total RNA was extracted using Nucleospin RNA extraction II Kit (740,955.250, Macherey–Nagel, Düren, Germany), or 500 µl TRIzol^™^, both, following the manufacturer’s protocol. RNA concentrations were measured with a NanoDrop1000 spectrophotometer (Thermo Fisher Scientific). Finally, first-strand cDNA was synthesized from 1 µg of total RNA using the NZY Reverse Transcriptase (NZYTech) and random primers or oligo(dT) primers (NZYTech), according to the manufacturer's instructions. A control “no RT” reaction (without reverse transcriptase enzyme) was performed in parallel to verify that all genomic DNA was degraded.

### Reverse transcription-coupled to quantitative PCR (RT-qPCR)

cDNA generated by reverse transcription was used as a template to perform the qPCR using gene-specific primers for PCR (Supplementary Table 1), and SYBR^®^ Green PCR Master Mix (Applied Biosystems, Waltham, MA, USA), following the manufacturer’s instructions, using the ABI75000 Sequence Detection System (Applied Biosystems). The relative mRNA levels for each gene were compared to those of *GAPDH* (endogenous control) and calculated by applying the comparative Ct method (2^−ΔΔCt^) [29]. To do so, amplification efficiencies were calculated for each set of primers performing cDNA serial dilutions.

### Semi-quantitative RT-PCR

Semi-quantitative RT-PCR was employed to check TUT4 gene silencing when needed. cDNA synthesized by reverse transcription was used as a template to quantify relative mRNA expression levels of TUT4, with GAPDH as an internal control (primers in Supplementary Table 1) using AmpliTaq^®^ DNA Polymerase (N8080160, Thermo Fisher Scientific), as instructed by the manufacturer. Cycler conditions were as follows: initial denaturation at 95 °C for 5 min; 28 cycles (or 25 for GAPDH) of 95 °C for 30 s (denaturation), 62 °C for 45 s (annealing), and 72 °C for 45 s (extension); final extension at 72 °C for 10 min. PCR amplifications were analyzed by gel electrophoresis placing 10 µL of each PCR product in a 2% agarose gel (w/v) in Tris–Borate-EDTA (TBE) 1 × stained with ethidium bromide. Then, band densities were quantified using ImageJ software. Three serial dilutions were prepared for the control silencing condition (LUC) to build a standard calibration curve and determine relative mRNA amounts in other experimental conditions.

### RNA-seq analysis

For the RNA-seq experiment, total RNA was extracted from SW480 cells treated with siRNAs targeting either LUC, DIS3L2 or DIS3L2 + TUTs. The assessment of RNA integrity was performed with an Agilent 2100 Bioanalyzer system (Agilent Technologies, San Diego, CA). RIN values were above 8 for all samples. 4 µg of total RNA per replicate were sent to Stab Vida (Lisbon, Portugal) for sample preparation and sequencing. RNA libraries were prepared from three independent biological replicates for each condition and enriched for long RNAs (> 200 nucleotides) by poly(A) selection. Next, mRNA was fragmented and converted to first strand cDNA using reverse transcriptase and random primers. Then, libraries were run on an Illumina HiSeq 2500 sequencing platform generating around 20 million paired end reads with an average read length of 282 base pairs (Supplementary Table 2). The raw fastq files are available at the European Nucleotide Archive through study accession number PRJEB59356.

Following quality control, each fastq file was processed with in-house Perl scripts [30] to remove the first 10 base pairs from each read and low quality reads. Adapter sequences were removed using Trimmomatic [31]. The processed reads were aligned to the human reference genome (ENSEMBL GRCh38.p10 version) using STAR aligner [32] to yield on average 84.71%, 84.41% and 84.11% uniquely mapped reads for LUC KD, DIS3L2 KD and DIS3L2 + TUTs KD, respectively (Supplementary Table 2).

Differentially expressed genes (DEGs) were identified by analyzing read counts per gene across all experimental conditions using the DESeq2 Bioconductor package (v.1.20.0), which model count data with negative normal distributions [33]. The counts were normalized to the library size, using the rlog method. Gene ontology (GO) analysis of DEG was performed using the R package, clusterProfiler from Bioconductor [34]. KEGG canonical pathways were used for pathway enrichment analysis.

### Western blot analysis

Protein extracts were obtained from lysis of transiently transfected cells. Cells were washed with 1 mL of pre-chilled PBS 1×, lysed in 100 µL of NP40 buffer supplemented with Proteiase inhibitor cocktail 2 (AppliChem GmbH, Germany) and centrifuged at 12,000 rpm, 4 °C for 5 min. Supernatants were collected and denatured with 1× (v/v) sodium dodecyl sulfate (SDS) sample buffer (NZYTech) at 95 °C for 10–20 min. After denaturation, the corresponding volume to 30 µg of total protein was loaded in an 8, 10 or 12% acrylamide/ bisacrylamide gel, resolved by SDS–polyacrylamide gel electrophoresis (SDS-PAGE) and transferred to methanol pre-activated polyvinylidene difluoride (PVDF) membranes (Bio-Rad, Hercules, CA, USA). Then, membranes were blocked for 1 h in blocking buffer [TBS-0.5% TW20: 150 mM NaCl, 50 mM Tris–HCl pH 7.5, 5% non-fat dried milk, and 0.5% (v/v) Tween 20]; or [TBS-0.5% Triton: 150 mM NaCl, 50 mM Tris–HCl pH 7.5, 5% bovine serum albumin (BSA), and 0.5% (v/v) Triton] at room temperature. After blocking, membranes were incubated overnight, at 4 °C, with the following primary antibodies diluted in blocking buffer: mouse anti-α-tubulin (T5168, Sigma-Aldrich) diluted at 1:50,000, rabbit anti-DIS3L2 (NBP2-38,264, Novus Biologicals) diluted at 1:500, rabbit anti-TUT7 (HPA020620, Sigma-Aldrich) diluted at 1:250, rabbit anti-TUT4 (HPA027973, Sigma-Aldrich) diluted at 1:250, rabbit anti-AZGP1 (HPA012582, Sigma-Aldrich) diluted at 1:250, mouse anti-mTOR (sc-517464, Santa Cruz) diluted at 1:400 rabbit anti-FRAP1 (SAB4300473, Sigma) diluted 1:250, rabbit anti-cyclin D1 (sc-718, Santa Cruz) diluted 1:250, and mouse anti-p-mTOR (sc-293133, Santa Cruz) diluted at 1:250. Then, membranes were washed 3–4 times for 5 min with washing buffer (TBS-0.5% TW20 or TBS-0.5% Triton) at room temperature. Immediately after washing, the detection was performed with the following secondary antibodies: peroxidase-conjugated anti-mouse IgG (Bio-Rad) diluted 1:4000 or anti-rabbit IgG (Bio-Rad) diluted 1:3000 in blocking buffer for 1 h at room temperature, followed by enhanced chemiluminescence (SuperSignal west Pico PLUS Chemiluminescent Substrate: Thermo Scientific, IL, USA).

### MTT assay

The 3-(4,5-dimethylthiazol-2-yl)-2,5-diphenyltetrazolium bromide (MTT) assay was employed to evaluate DIS3L2 impact on cell viability. Cells were seeded in 96-well plates at different densities depending on the cell type and incubation time in order to have a logarithmic growth phase (Supplementary Table 3). For gene silencing, 24 h after seeding, cells were transiently transfected with 5 pmol of siRNAs. For overexpression studies, cells were transiently transfected 24 h after seeding, with 38 ng of each plasmid. Forty-eight and 72 h post-transfection, cells were washed twice with 100 µL PBS 1× and treated with 100 µL of MTT reagent (M2128, Sigma-Aldrich, St. Louis, MO USA). MTT was first prepared as a stock solution of 5 mg/mL in PBS 1× (10,010,023, Gibco, Thermo Fisher Scientific) and filtered. Then, before cell treatment, MTT was dissolved in the corresponding medium at a final concentration of 0.5 mg/mL. After incubation for 3 h at 37 °C, MTT solution was removed and 100 µL of solubilizing buffer (Dimethyl sulfoxide; Sigma-Aldrich) were added to each well in order to dissolve the remaining formazan crystals. After a 30-min incubation at room temperature, protected from light with shaking at 800 rpm, the absorbance was measured by a spectrophotometric plate reader (VersaMax^™^ Absorbance Microplate Reader) at 570 nm to determine cell viability.

### Scratch wound-healing assay

The wound-healing assay was used in order to evaluate DIS3L2 contribution to cell migration. For gene silencing, cells were seeded in 6-well plates (NCM460: 3.75 × 10^5^ cells/mL; SW480: 2.5 × 10^5^ cells/mL), and 24 h after, transiently transfected with 250 pmol of siRNAs targeting DIS3L2 or LUC (control condition). For overexpression studies, cells were seeded in 6-well plates (SW480: 3.5 × 10^5^ cells/mL; NCM460: 5 × 10^5^ cells/mL), and 24 h after, transiently transfected with 980 ng of selected plasmids. Forty-eight hours post-transfection cells were grown to saturating confluence. Then, across the center of the well, a “scratch” was gently created on the cell monolayer in a straight line, using a 200 µL pipette tip. In order to assess cell migration without interferences from cell proliferation, cells were pre-treated with mitomycin C (1 µg/mL), 4 h before scratching. Immediately after scratching, cells were washed with PBS (10,010,023, Gibco, Thermo Fisher Scientific) to remove detached cells and replaced with fresh medium supplemented with 10% FBS (Gibco, Thermo Fisher Scientific). During the whole wound-healing experiment, cells were maintained in an incubator at 37 °C, in a humidified atmosphere with 5% (v/v) CO_2_. A phase-contrast microscope with a 10× objective was used to obtain images of cell migration at 0, 24, 48 and 72 h after scratching. Captured images were analyzed using ImageJ software. Cell-free areas were traced to calculate the wound area. At 0 h of incubation, the gap created by the wound was calculated and considered 100% of the wound area. The subsequent areas at different time points were quantified and represented relatively to the initial gap area.

### Culture-insert wound-healing assay

Wound-healing assays using culture-inserts were used for HCT116, Caco-2 and HT-29 cells due to technical difficulties to apply the scratch wound-healing assay to these cell lines. Cells were seeded in culture-insert wells, pre-inserted into µ-Dish 35 mm (81,176, Ibidi, Martinsried, Germany). Seeding cell densities were optimized for each cell type (Supplementary Table 4). For gene silencing, 24 h after seeding, cells were transiently transfected with 6 pmol of siRNAs. For overexpression studies, cells were transiently transfected 24 h after seeding, with 42 ng of each plasmid. Forty-eight h post-transfection, cells reached 100% confluence and culture-inserts were removed using sterile tweezers creating a cell-free gap of 500 µm. Again, to prevent interference from cell proliferation, cells were pre-treated for 4 h with mitomycin C (1 µg/mL) before insert removal. Then, wells were filled with fresh medium supplemented with 10% FBS (Gibco, Thermo Fisher Scientific). Images of cell migration into the gap were acquired using a phase-contrast microscope with a 10 × objective at 0, 24, 48 and 72 h after insert removal. Then, images were analyzed using ImageJ software to measure the remaining gap area at each time point.

### Transwell migration and invasion assay

The transwell assay was used to track both, cell migration and invasion. This technique uses a 24-well chamber with a lower and an upper compartment separated by a Polyester (PET) membrane containing pores of 8 µm (10,769–242, VWR, Pennsylvania, USA). Cells were seeded in 6 well-plates (NCM460: 3.75 × 10^5^ cells/mL; SW480: 2.5 × 10^5^ cells/mL; HCT116: 2.5 × 10^5^ cells/mL; HT-29: 4 × 10^5^ cells/mL), and after 24 h, were transiently transfected with 250 pmol of siRNAs targeting DIS3L2 or LUC (control condition). Twenty-four h post-transfection, cells were trypsinized and a cell suspension of 2.5 × 10^5^ cells/mL was prepared in fresh medium supplemented with 1% FBS (Gibco, Thermo Fisher Scientific). Then, 300 µL (7.5 × 10^3^ cells) were placed in the upper chamber. In order to examine cell invasion, 100 µL of diluted Matrigel (356,237, Corning, NY, USA) at a final concentration of 2 mg/mL were placed in the upper chamber at least 2 h before placing cells on top. The lower chamber was filled with 600 µL of fresh medium supplemented with 20% FBS (Gibco, Thermo Fisher Scientific). Cells were then incubated for 48 h at 37 °C, in a humidified atmosphere with 5% (v/v) CO_2_. Next, membranes were fixed with 4% formaldehyde for 10 min, permeabilized with methanol for 10 min and stained with 0.1% crystal violet for 20 min. After every step, membranes were always washed twice with PBS 1×. Subsequently, non-moved cells were removed from the upper surface with cotton swabs. At least five randomly selected images of the lower side of the membrane were captured using a phase-contrast microscope with a 10 × objective. Then, images were analyzed and the number of migrated or invaded cells was quantified using ImageJ software.

### Statistical analysis

All statistical tests were performed in R v4.2.1. When population data were normally distributed, two-tiled, unpaired t tests were used to compare the means of single control groups to single test groups. The Mann–Whitney U test (also called Wilcoxon rank-sum test) was applied for non-normal variables. If multiple comparisons were required, a one-way ANOVA was performed. Gene expression data handled with DESeq2 package was statistically analyzed using a Wald test, which creates lists of DEGs with adjusted p values using Benjamini–Hochberg procedure [35]. All experiments had at least three independent replicates and results are visualized as mean ± standard error of the mean (SEM). Statistical significance was represented as follows: p < 0.05 (*), p < 0.01 (**) and p < 0.001 (***).

## Results

### DIS3L2 is overexpressed and predicts a poor prognosis in CRC

**Fig. 1:**
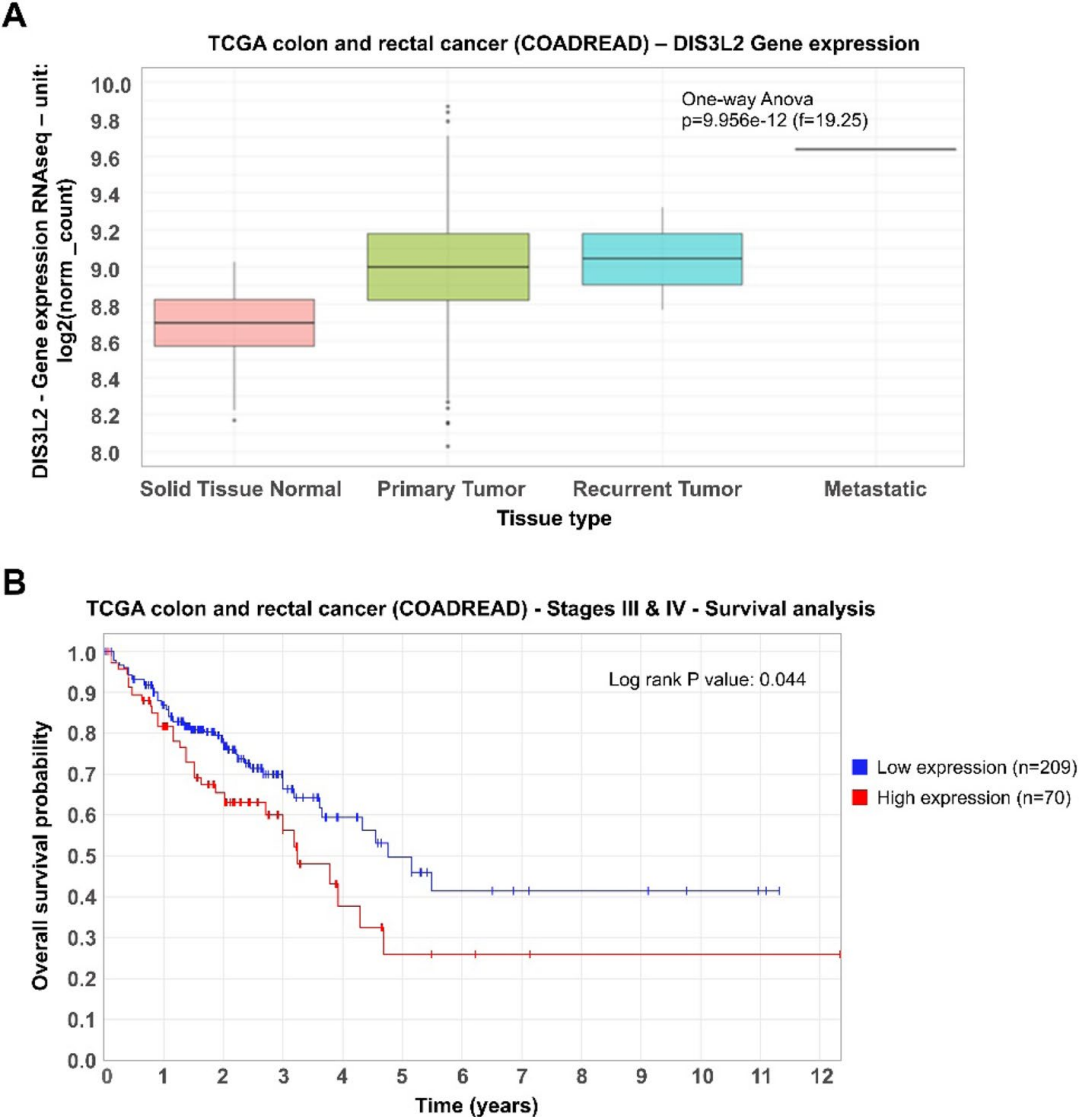
DIS3L2 is overexpressed in colorectal cancer (CRC) tissues and its upregulation correlates with poorer prognosis in patients with advanced CRC (stages III and IV). **A** Boxplot represents DIS3L2 mRNA levels in CRC tissues versus non-tumorigenic colonic samples, according to transcriptomic datasets from The Cancer Genome Atlas (TCGA). **B** Kaplan–Meier plot for high versus low DIS3L2 gene expression in patients affected by CRC (cut-off value: 3.2 FPKM), according to clinical and transcriptomic data from TCGA database

To explore the clinical significance of DIS3L2 in cancer, we decided to investigate its gene expression across different tumorigenic tissues. We performed bioinformatic analysis of DIS3L2 mRNA levels using the public RNA sequencing datasets from The Cancer Genome Atlas (TCGA). RNA-seq gene expression data from tumor tissues and matched normal samples was used to evaluate DIS3L2 expression. We observed that DIS3L2 was significantly overexpressed in colorectal cancer tissues compared to non-tumorigenic tissues (p < 0.001, Fig. 1A).

Next, we decided to analyze the prognostic value of DIS3L2 in patients with colorectal cancer using RNA-seq and clinical data from TCGA database. To perform survival analysis and generate Kaplan–Meier plots, we used the R packages Survival (3.3.1) and Survminer (v.0.4.9). Interestingly, results show that high DIS3L2 gene expression correlates with poorer prognosis in patients with advanced CRC (stages III and IV) (cut-off value: 3.2 FPKM; p < 0.05, Fig. 1B). Altogether, these results reflect a potential pro-tumorigenic role for DIS3L2 upregulation in CRC.

### RNA deep-sequencing shows that DIS3L2 heavily impacts the transcriptome of SW480 cells

**Fig. 2:**
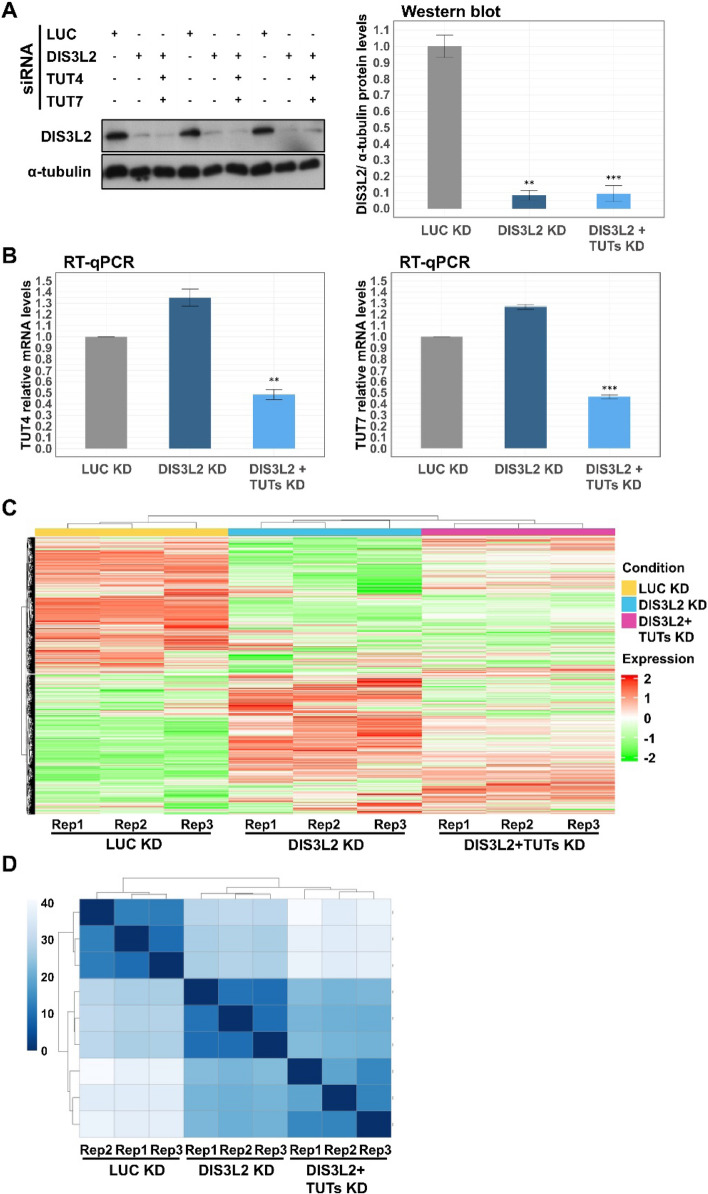
Knockdown (KD) of DIS3L2 largely affects the transcriptome of SW480 cells. **A** Western blot analysis for DIS3L2 and α–tubulin (loading control) to monitor DIS3L2 KD efficiencies from lysates used in the RNA-seq sample preparation; bar plot represents DIS3L2 protein levels after normalizing to α–tubulin, and to siLUC-treated cells (control condition), arbitrarily set to 1. **B** TUT4 (on the left) and TUT7 (on the right) mRNA levels normalized to glyceraldehyde-3-phosphate dehydrogenase (GAPDH; internal control) mRNA and determined by RT-qPCR to check TUTs KD efficiencies from lysates used in the RNA-seq sample preparation. Bar plots represent fold-change of each gene in each experimental condition, relative to mRNA levels from siLUC-treated cells, arbitrarily set to 1. **C** Heatmap of all genes detected by the RNA-seq, with a minimum of 11 counts in at least one sample. Genes are represented in the vertical axis, conditions in the horizontal axis and expression values are displayed as a Z-score (mean = 0) across samples. Color saturation represents the magnitude of deviation from the median (green and red color saturation correspond to values that are lower and greater than the row mean, respectively). **D** Sample-to-sample distance heatmap calculated after estimation of stabilized variance of normalized counts of gene expression across replicates. Square color saturation indicates relationship between transcriptomes of all RNA-seq samples. n = 3, asterisks (*) indicate statistical significance relative to protein levels (A) or mRNA levels (B) in control conditions (LUC KD): p < 0.05 (*), p < 0.01 (**) and p < 0.001 (***)

Several studies have described the crucial role of DIS3L2 and uridylation in cytoplasmic RNA surveillance, promoting decay of miRNAs, mRNAs and ncRNAs [9, 36–38], and it has been documented that DIS3L2 dysfunction disrupts transcriptome homeostasis [6]. Here, we investigated what would be the transcriptome-wide impact of decreasing the expression of TUTs 4 and 7 and/or DIS3L2 in a colorectal tumorigenic context. To do so, we performed a high-throughput RNA-sequencing experiment in colorectal cancer SW480 cells depleted from DIS3L2 or DIS3L2 + TUTs, by siRNA treatment. As control, we used an siRNA targeting the exogenous transcript of firefly luciferase (siLUC). Knockdown efficiencies were verified in three independent replicates by Western blot for DIS3L2 and by RT-qPCR for TUTs (Fig. 2A–B, Supplementary Table 5). Total RNA was isolated and subjected to library preparation and paired-end sequencing. Then, sequencing fastq files were processed and aligned to the human reference genome (ENSEMBL GRCh38.p10 version). Alignment data details are shown in Supplementary Table 2.

Only genes with a minimum of 11 read counts in at least one sample (threshold to define “expressed” and “unexpressed” genes) were considered for downstream analysis. Differentially expressed genes (DEGs) were identified by analyzing the counts per gene across experimental conditions through the DESeq 2 package [33]. DESeq2 normalizes the data matrix of counts per gene by applying a regularized-logarithm transformation (rlog) that transforms the raw data to a logarithmic scale. Normalized gene datasets were subjected to principal component analysis (PCA) based on normalized expression values that are estimated by the number of reads mapping a locus (transcript) normalized to the length of the locus and density of the dataset. The PCA plot (Supplementary Fig. 1) shows highest degrees of variability between control (siLUC) and DIS3L2 KDs, as demonstrated by the distant clustering on the principal component (PC) 1 (87% variance). Importantly, replicates from same condition clustered the closest, while the independent experimental conditions are separated from each other, which reflects that the greatest source of variation arises from the three experimental conditions used.

A heatmap plot clustering all genes detected in the RNA-seq by their gene expression patterns revealed robust differences between the three conditions (Fig. 2C), with DIS3L2 and DIS3L2 + TUTs KDs displaying higher correlation with each other as expected. The major impact over the transcriptome landscape was observed in DIS3L2-depleted cells in comparison with gene expression patterns of control condition (Fig. 2C). A sample-to-sample heatmap was also computed to visualize overall gene expression between experimental conditions and transcriptomic similarities among them. As represented in the dendrogram of Fig. 2D, replicates from each condition cluster together as expected. Moreover, DIS3L2 + TUTs KD showed a closer relationship with the control condition than single depletion of DIS3L2, which suggests that the latter has a more divergent transcriptome.

**Fig. 3:**
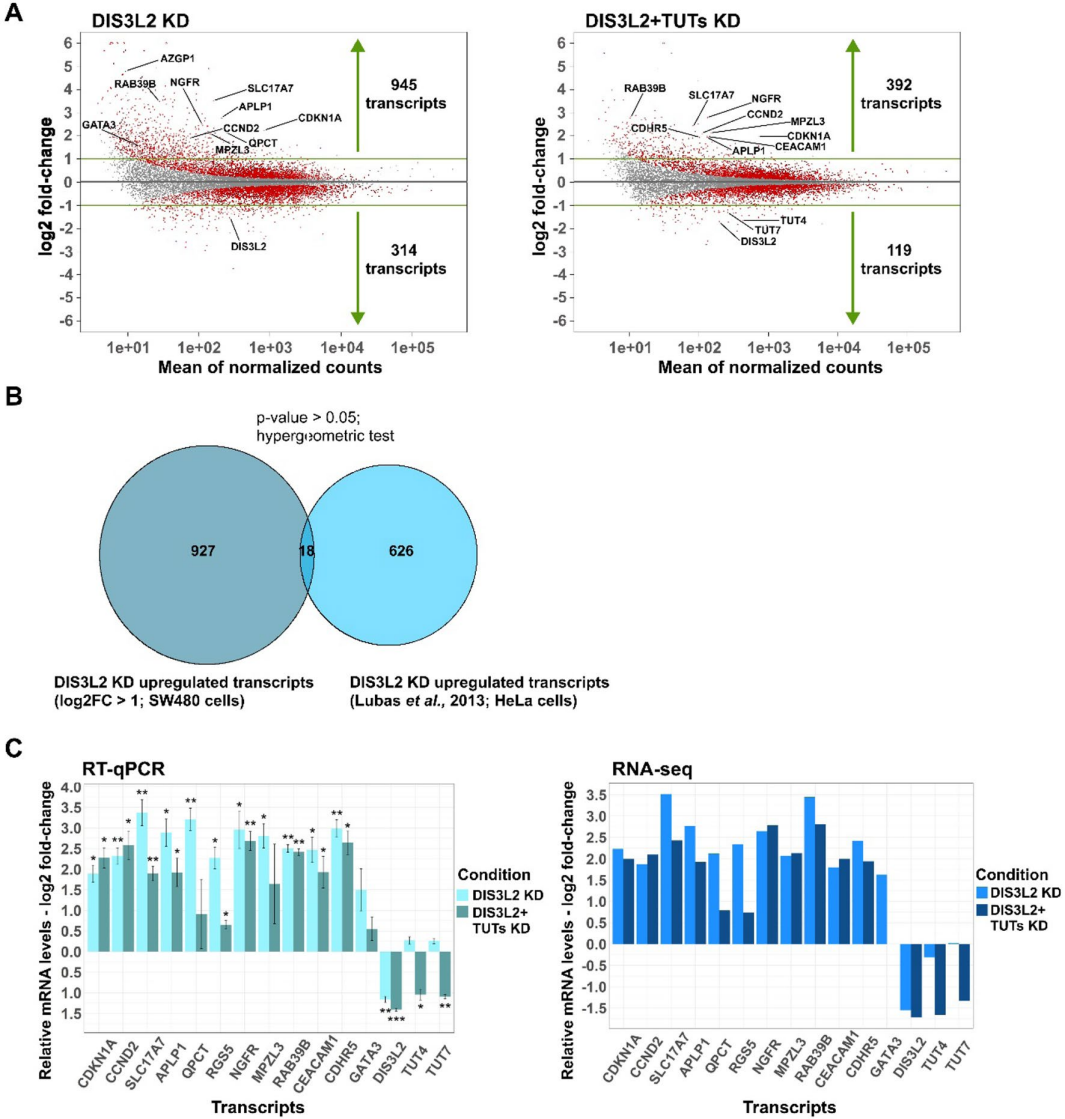
Depletion of DIS3L2 leads to more differentially expressed genes (DEGs) than DIS3L2 + TUTs knockdown (KD). **A** MA plot for visual representation of gene expression changes when comparing LUC KD (control condition) versus either DIS3L2 KD (left) or DIS3L2 + TUTs KD (right). Differentially expressed genes (DEGs) identified by DESeq 2 with adjusted p value < 0.05 (Wald test) are represented as red points. **B** Overlap analysis of DIS3L2-dependent mRNA targets obtained from our RNA-seq in SW480 cells (left circle) and from Lubas et al. transcriptomic analysis in HeLa cells (right circle). The overlap between circles represents the number of transcripts that are upregulated upon DIS3L2 knockdown (KD) in both gene sets (18). Statistical significance of this overlap is estimated by a hypergeometric test (p value < 0.05). **C** Validation of RNA-seq experiment; comparison of mRNA fold-changes of a set of selected transcripts (CDKN1A, CCND2, SLC17A7, APLP1, QPCT, RGS5, NGFR, MPZL3, RAB39B, CEACAM1, CDHR5, GATA3, DIS3L2, TUT4 and TUT7) by RT-qPCR (left) and RNA-seq (right). n = 3, asterisks (*) indicate statistical significance relative to mRNA levels in control conditions (LUC KD): p < 0.05 (*), p < 0.01 (**) and p < 0.001 (***)

Transcriptomes from each knockdown condition were compared with the control condition to identify DEGs as outlined in the DESeq 2 package. Differences were considered statistically significant with adjusted p values < 0.05. Detected DEGs were listed based on their log2 fold-change (FC) (Supplementary file 1). We identified a total of 1259 (945 upregulated and 314 downregulated with |log2FC|> 1) DEGs after depletion of DIS3L2 (Fig. 3A). In contrast, this number was lower upon DIS3L2 + TUTs depletion (511 DEGs, of which 392 upregulated and 119 downregulated with |log2FC|> 1) (Fig. 3A). Considering the number of deregulated genes, these results suggests that single depletion of DIS3L2 had a bigger impact over the transcriptome than the triple knockdown. We further examined a previous set of DIS3L2 targets (680 transcripts) identified in HeLa cells by Lubas and colleagues [6]. After cross-referencing this gene set with our pool of significant upregulated transcripts after DIS3L2 KD, we found only 18 targets in common (p value > 0.05, hypergeometric test; Fig. 3B).

Next, a set of 13 upregulated genes with log2FC > 1.5 in at least one of the two KD conditions were selected for RNA-seq validation by independent RT-qPCR analysis on independent samples. Figure 3C shows that changes in gene expression of selected targets were similar between the two sets of samples in spite of the different methods used, validating our transcriptome profiling. Furthermore, this set of upregulated transcripts was subjected to gene co-expression analysis with DIS3L2 using transcriptomic data from the TCGA database. Such analysis was performed on tumorigenic samples from patients with advanced CRC (stages III and IV). Our results show that 9 out of 12 genes exhibited a significant negative correlation with DIS3L2 mRNA expression levels, while none of them showed a significant positive correlation (Supplementary Fig. 2).

**Fig. 4:**
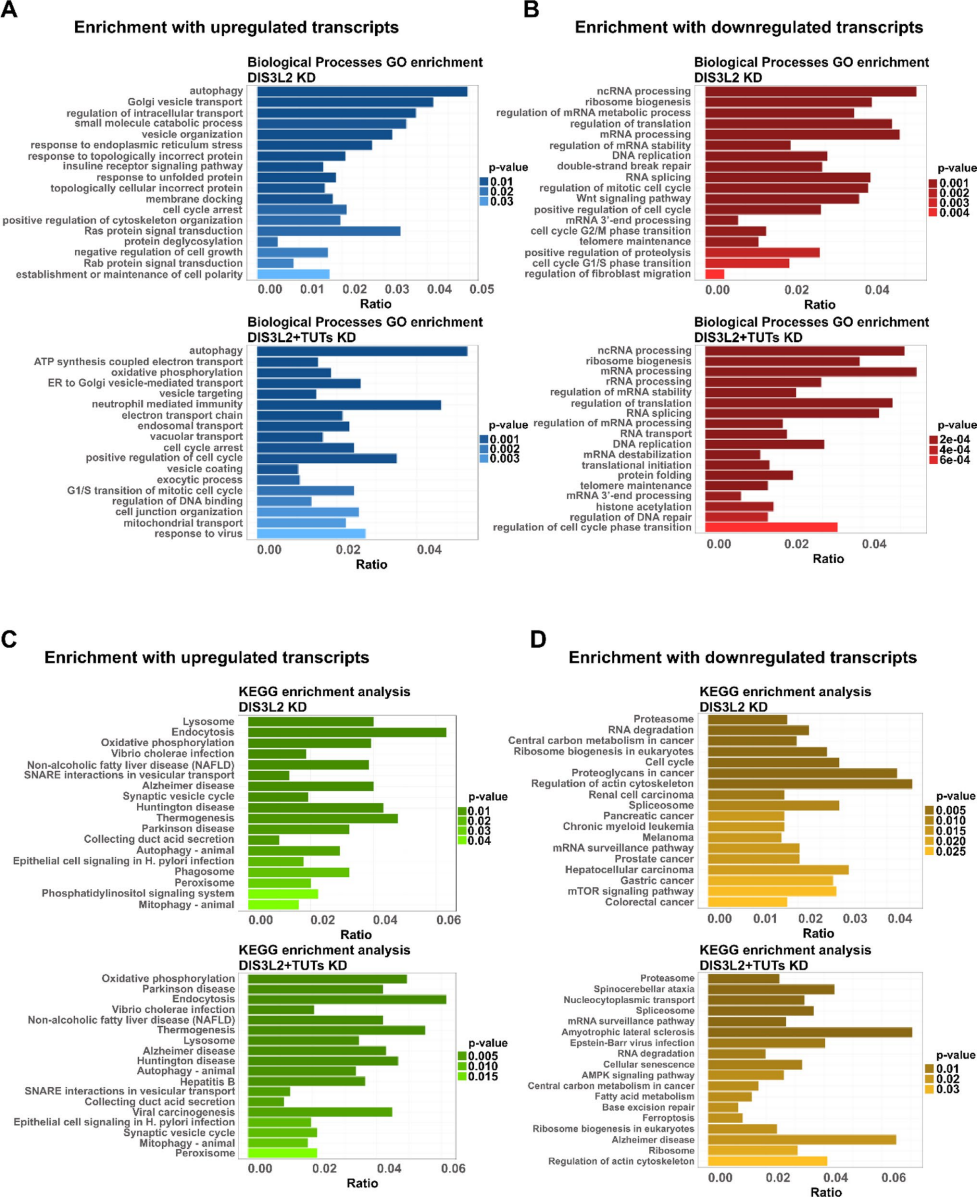
Upregulated mRNAs after depletion of DIS3L2 or DIS3L2 + TUTs, are enriched in GO terms associated with cell cycle regulation. **A** Histograms show gene ontology (GO) analysis of biological processes enriched in the pool of upregulated transcripts either after DIS3L2 knockdown (KD) (top) or after DIS3L2 + TUTs KD (bottom). **B** GO enrichment for biological processes in the pool of downregulated transcripts either after DIS3L2 KD (top) or after DIS3L2 + TUTs KD (bottom). **C** Histogram displaying KEGG pathways enriched in the pool of upregulated transcripts after DIS3L2 KD (top) and DIS3L2 + TUTs KD (bottom). **D** KEGG pathways enrichment in the pool of downregulated transcripts either after DIS3L2 KD (top) or after DIS3L2 + TUTs KD (bottom). GO and KEGG terms are ranked by p value; ratio in x-axis of all plots is calculated by the number of transcripts enriched for each GO/KEGG term versus total number of up- or downregulated genes for each KD condition, respectively

Gene ontology analysis was conducted over the pool of significantly (adjusted p value < 0.05) deregulated transcripts in DIS3L2- and DIS3L2 + TUTs-depleted cells. GO terms for biological processes were enriched in key steps of the secretory pathway and autophagy-related terms for upregulated genes from both KD conditions, including endoplasmic reticulum and Golgi apparatus-dependent transport (Fig. 4A). Interestingly, we also observed an enrichment in genes associated with GO terms linked to negative regulation of cell proliferation, such as “cell cycle arrest” and “negative regulation of cell growth”. Conversely, GO analysis for the group of genes significantly downregulated by the same KD conditions showed enrichments in terms associated with the promotion of cell cycle progression (Fig. 4B). Notably, the downregulated gene group was also highly enriched in multiple GO terms related with cellular events known to be modulated by DIS3L2 activity (Supplementary file 2), such as “ncRNA processing”, “regulation of mRNA stability” and “RNA splicing”, among others (Fig. 4B).

KEGG pathway analysis from upregulated transcripts after DIS3L2 or DIS3L2 + TUTs depletion showed enrichment in membrane-limited organelles and associated processes, energy metabolism as well as neurodegenerative disorders (Fig. 4C). On the other hand, analysis of downregulated transcripts after knockdown of DIS3L2 showed enrichment in pathways associated with multiple types of cancers, namely “colorectal cancer”, including “regulation of actin cytoskeleton” and “mTOR signaling pathway” (Fig. 4D). In contrast, a clear loss in cancer related associations was observed upon KEGG pathway analysis of the transcripts downregulated in response to the triple knockdown (DIS3L2 + TUTs) (Fig. 4D).

### Depletion of DIS3L2 impairs cell viability of CRC cells

**Fig. 5:**
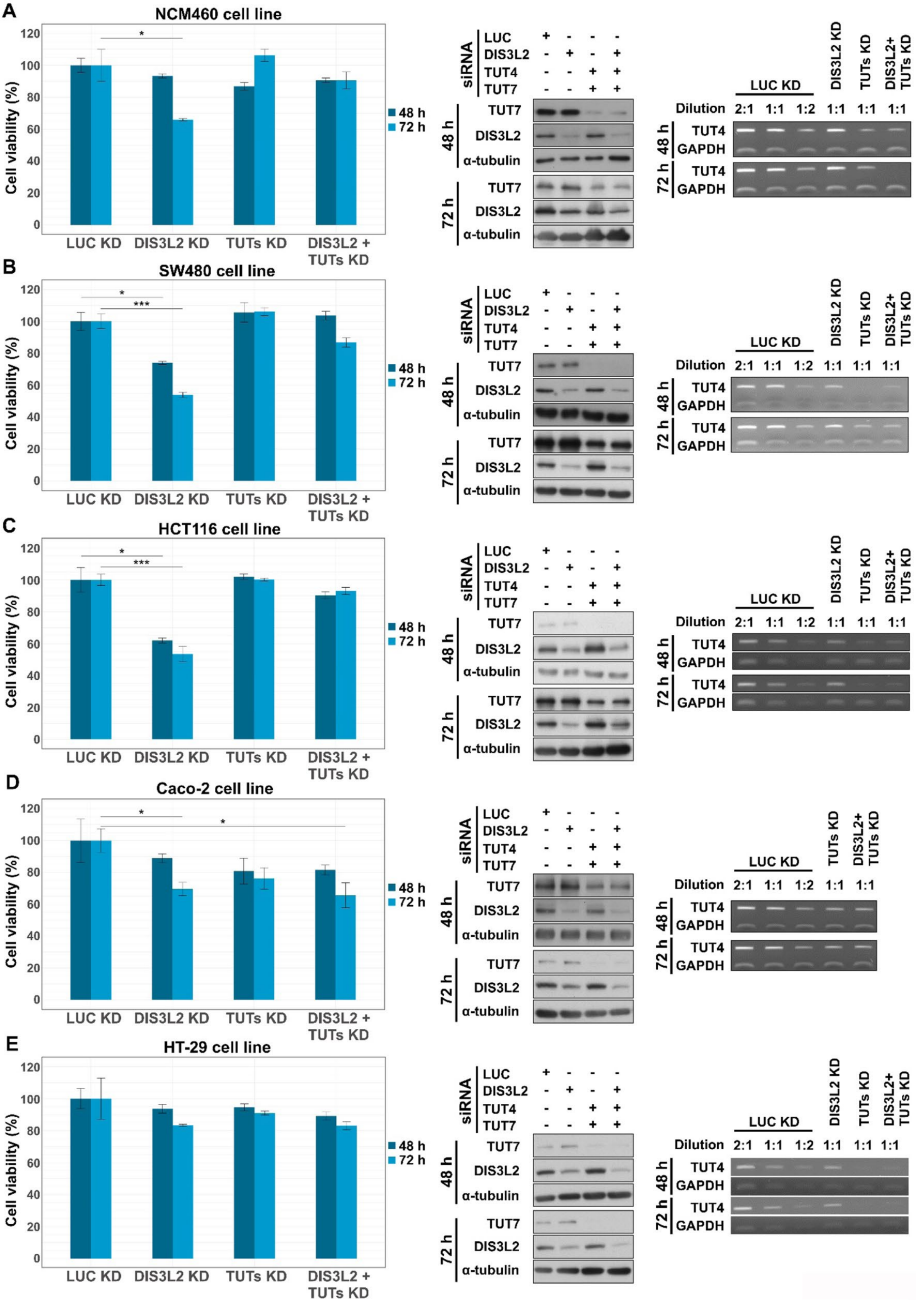
DIS3L2 knockdown (KD) significantly impairs cell viability of SW480 and HCT116 cell lines. Bar plots represent MTT assays performed in non-transformed NCM460 colonocytes (**A**) and colorectal cancer cell lines SW480 (**B**), HCT116 (**C**), Caco-2 (**D**) and HT-29 (**E**). MTT assays were performed 48 h and 72 h after mock transfection with siLUC (control condition) or transfection with siDIS3L2, siTUTs 4/7, or the double KD with siDIS3L2 + siTUTs 4/7. Representative Western blots and semi-qPCRs of KD efficiencies in all cell lines are displayed on the right side of each bar plot. α–tubulin and GAPDH were used as internal controls in Western blot and semi-qPCR analysis, respectively. n = 4, statistical significance relative to mock conditions are indicated as: (*) p < 0.05, (**) p < 0.01, and (***) p < 0.001

The functional annotation clustering analysis performed over the differential expressed genes guided us to investigate whether interference with DIS3L2 function and/or the uridylation machinery would affect the oncogenic properties of CRC cells, namely regarding cell proliferation and viability. Non-transformed colonic NCM460 cells (control cell line) and four CRC cell lines with different genetic backgrounds and tumorigenic properties (SW480, HCT116, Caco-2 and HT-29) were depleted of either DIS3L2, TUTs 4/7, or DIS3L2 + TUTs 4/7 by siRNAs. Again, non-specific siLUC was used as control. Cell viability was then monitored 48 h and 72 h after siRNA transfection via 3-(4,5-dimethylthiazol-2-yl)-2,5-diphenyltetrazolium bromide (MTT) assay, which detects differences in cell metabolic activity. Knockdown efficiencies were monitored by Western blot or semi-quantitative RT-PCR (Fig. 5, Supplementary Table 6).

Our results revealed that downregulation of DIS3L2 for 48 h did not have any significant impact on cell viability of non-tumorigenic NCM460 cells, although 72 h post-transfection cell viability decreased slightly (~ 30%) compared to siLUC treated cells (Fig. 5A). Knockdown of TUTs alone did not induce any significant impact on cell viability at any of the two time points. Interestingly, NCM460 cell viability at 72 h was also unaltered upon the double downregulation of DIS3L2 and uridylation. On the other hand, DIS3L2-depleted SW480 and HCT116 cells exhibited a significant decrease in cell viability already at 48 h post-transfection (by ~ 30% and ~ 40%, respectively) (Fig. 5B–C). Differences were even more pronounced at 72 h post-transfection, since DIS3L2 KD reduced cell viability to nearly 50% of siLUC control in either cell lines. In contrast, the triple knockdown of DIS3L2 and TUTs rescued the loss of cell viability in both SW480 and HCT116 cells, at either time points. Notably, as seen for NCM460 cells, interference with uridylation alone (siTUTs) did not affect the viability of either CRC cell lines (Fig. 5B–C). Also, as NCM460 cells, the less tumorigenic Caco-2 CRC cell line [39, 40] only experienced a mild impairment in cell viability after 72 h post-transfection of siDIS3L2 (DIS3L2 KD), while HT-29 cells, known to show low oncogenicity [41, 42], did not respond to any of the silencing approaches (Fig. 5D–E). These findings indicate that interference with DIS3L2 abundance can disturb cell viability, but the effect appears to be more pronounced in more oncogenic (SW480 and HCT116) than in better differentiated cell lines (Caco-2, HT-29 and non-transformed NCM460). In contrast, while interference with TUTs does not seem to affect cell viability, co-interference with uridylation and DIS3L2 can apparently attenuate the impact of DIS3L2 KD on cell viability.

Next, we assessed the effects of overexpressing wild type DIS3L2 in the viability of our model cell lines. Overexpression efficiencies were assessed by Western blot (Supplementary Fig. 3). Since we observed an important reduction of cell viability in SW480 and HCT116 cells, we expected to see the opposite effect when overexpressing DIS3L2. Interestingly, in contrast to the depletion experiments, DIS3L2 overexpression did not significantly change the viability of any of the cell lines, including that of SW480 and HCT116 cells that were significantly affected by DIS3L2 depletion (Supplementary Fig. 3). We therefore tested whether increased uridylation was required to enable DIS3L2 overexpression to affect cell viability. SW480 and HCT116 cells were transfected simultaneously with plasmids encoding for TUT4, TUT7 and DIS3L2. Again, no significant differences were observed in cell viability compared to mock transfected (pcDNA3.1 +) control conditions. These results indicate that overexpression of TUTs 4/7 does not further sensitize cells to increased expression of DIS3L2 (Supplementary Fig. 4).

### DIS3L2 knockdown inhibits the mTOR signaling pathway

**Fig. 6:**
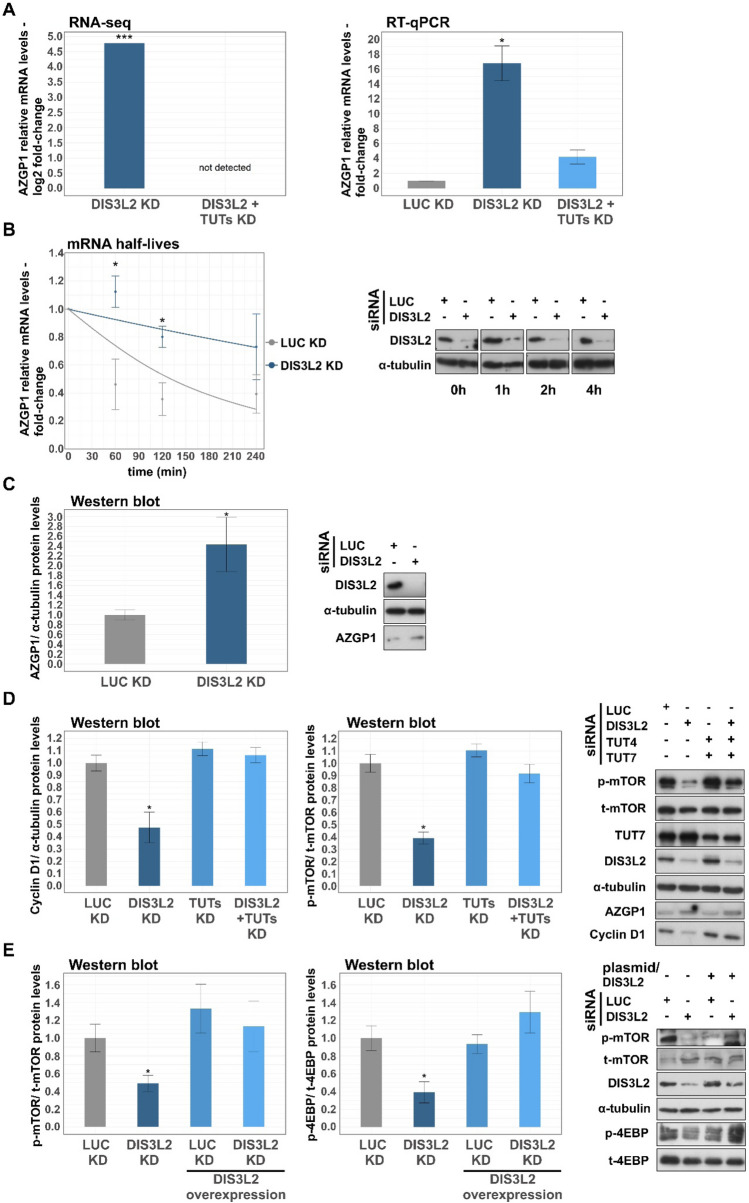
Loss of DIS3L2 expression upregulates AZGP1 mRNA and protein levels and inhibits the mTOR signaling pathway. **A** AZGP1 mRNA levels normalized to glyceraldehyde-3-phosphate dehydrogenase (GAPDH; internal control) mRNA and determined by RNA-seq (left) and RT-qPCR (right) in SW480 cells treated with siRNAs targeting LUC (control condition), DIS3L2 or DIS3L2 + TUTs. **B** mRNA stability of AZGP1 in SW480 cells transfected with siRNAs targeting LUC (control condition) and DIS3L2. The mRNA levels were determined by RT-qPCR at various time points (0, 60, 120, 240 min) after DRB treatment. AZGP1 mRNA decay rates were plotted by normalizing the mRNA level of each time point to that of 0 h in each condition; statistical significance is relative to mock condition at each time point. **C** Bar plot representing AZGP1 protein levels determined by Western blot in siLUC- and siDIS3L2-treated SW480 cells. On the right, a representative Western blot analysis for DIS3L2, AZGP1 and α–tubulin (loading control). **D** Bar plots representing Cyclin D1 and p/t-mTOR protein levels determined by Western blot in SW480 depleted from DIS3L2 or DIS3L2 + TUTs, after normalizing to protein levels of siLUC-treated cells. Representative Western blot analysis for phospho-Ser2448-mTOR (p-mTOR), total-mTOR (t-mTOR), TUT7, DIS3L2, AZGP1, Cyclin D1 and α–tubulin (loading control) to monitor the impact of DIS3L2 depletion in the mTOR signaling pathway in SW480 cells. **E** Bar plots representing p/t-mTOR and p/t-4EBP protein levels determined by Western blot in SW480 cells treated with siRNAs targeting LUC (control condition) or DIS3L2 with or without DIS3L2 overexpression. Representative Western blot analysis for p-mTOR, t-mTOR, DIS3L2, phosphor-Ser-112-4EBP (p-4EBP), total-4EBP (t-4EBP) and α-tubulin. n ≥ 3, statistical significance relative to mock condition are indicated as: (*) p < 0.05, (**) p < 0.01, and (***) p < 0.001

The mTOR signaling pathway, one of the enriched pathways in our KEGG analysis for upregulated DEGs after DIS3L2 KD, is a key regulator of cell viability and growth, sensing and integrating diverse nutritional and environmental cues [43]. Noticing that AZGP1, a known endogenous inhibitor of the mTOR pathway [44–46], was highly upregulated after DIS3L2 depletion in our RNA-seq experiment (> tenfold; Fig. 6A), we investigated the potential role of DIS3L2 in the mTOR pathway. In order to validate the results from RNA-seq, we firstly measured AZGP1 mRNA levels by RT-qPCR in SW480 cells depleted from DIS3L2 or DIS3L2 + TUTs. As represented in Fig. 6A, RT-qPCR analysis shows that AZGP1 was significantly upregulated (~ 17-fold) after DIS3L2 KD, comparing to control conditions (siLUC-treated cells). However, AZGP1 was no longer upregulated after the triple KD (siDIS3L2 + siTUT4/7), similar to that observed in the RNA-seq data (Fig. 6A). Next, we analyzed the mRNA half-live of AZGP1 in SW480 cells treated with siRNAs targeting LUC or DIS3L2 (Fig. 6B). Forty-eight hours post-transfection, transcription was inhibited by the addition of DRB and AZGP1 mRNA levels were measured by RT-qPCR at several subsequent time points (0, 60, 120 and 240 min). Knockdown efficiencies for each time point were verified by Western blot analysis (Fig. 6B, Supplementary Table 7). In addition, mRNA levels from ATF3, a known DIS3L2-target (positive control) and BAG1, a DIS3L2-resistant transcript (negative control) [9], were monitored by RT-qPCR (Supplementary Fig. 5). Results show that AZGP1 mRNA half-life upon DIS3L2 depletion was significantly higher than that in siLUC-treated cells, indicating that AZGP1 is a direct mRNA target of DIS3L2.

Next, we analyzed AZGP1 protein levels by Western blot in siLUC- and siDIS3L2-treated SW480 cells. As shown in Fig. 6C, loss of DIS3L2 results in a significant increase of AZGP1 protein expression. Having identified a notable upregulation of an mTOR signaling pathway suppressor, we asked if mTOR activity was inhibited upon DIS3L2 depletion. We assessed the activation state of the mTOR protein, by measuring its phosphorylation levels at Ser-2448. Interestingly, we detected a decreased phosphorylation of mTOR in DIS3L2 KD condition versus LUC KD condition that was clearly being translated downstream through the pathway, since it resulted in a significant decrease in cyclin D1 expression (Fig. 6D), a well-known target of the mTOR signaling pathway with a key role in G1/S cell cycle progression [47]. Remarkably, p-mTOR and cyclin D1 protein levels were no longer downregulated after triple KD of DIS3L2 + TUTs (Fig. 6D). In addition, we conducted a gene co-expression analysis between DIS3L2 and CCND1, the gene encoding cyclin D1, in advanced CRC samples (stages III and IV) using transcriptomic data from the TCGA database. Results show a significant positive correlation between DIS3L2 and CCND1 (Supplementary Fig. 6). In order to confirm that lower p-mTOR expression levels were attributed to the depletion of DIS3L2, we transfected SW480 cells with plasmids expressing DIS3L2, 48 h after treatment with siRNAs against LUC or DIS3L2 (Fig. 6E). Results indicate that the rescue of DIS3L2 expression levels in the DIS3L2 KD background led to a significant increase in levels of p-mTOR. Furthermore, we assessed the phosphorylation status of 4EBP, a well-known downstream player in the mTOR signaling pathway, and we observed significant lower levels of phosphorylated-4EBP (p-4EBP) after DIS3L2 KD. Interestingly, similar to p-mTOR, protein levels of p-4EBP were restored after DIS3L2 overexpression in SW480 cells. Altogether, these results indicate that depletion of DIS3L2 expression can lead to suppression of the mTOR signaling pathway in CRC cells.

### DIS3L2 depletion impairs motility in SW480 cells

Since another of the pathways enriched upon DIS3L2 depletion was “Regulation of the actin cytoskeleton”, and the DIS3L2 target, AZGP1, is known for its inhibitory effects in CRC cell migration [44], we next investigated whether the depletion or overexpression of DIS3L2 could alter the migratory behavior of our CRC cell lines. We first employed wound-healing assays, pre-treating cells with mitomycin C, a DNA synthesis inhibitor that serves as a proliferation inhibitor ensuring that any observed variations are only due to cell migration.

**Fig. 7:**
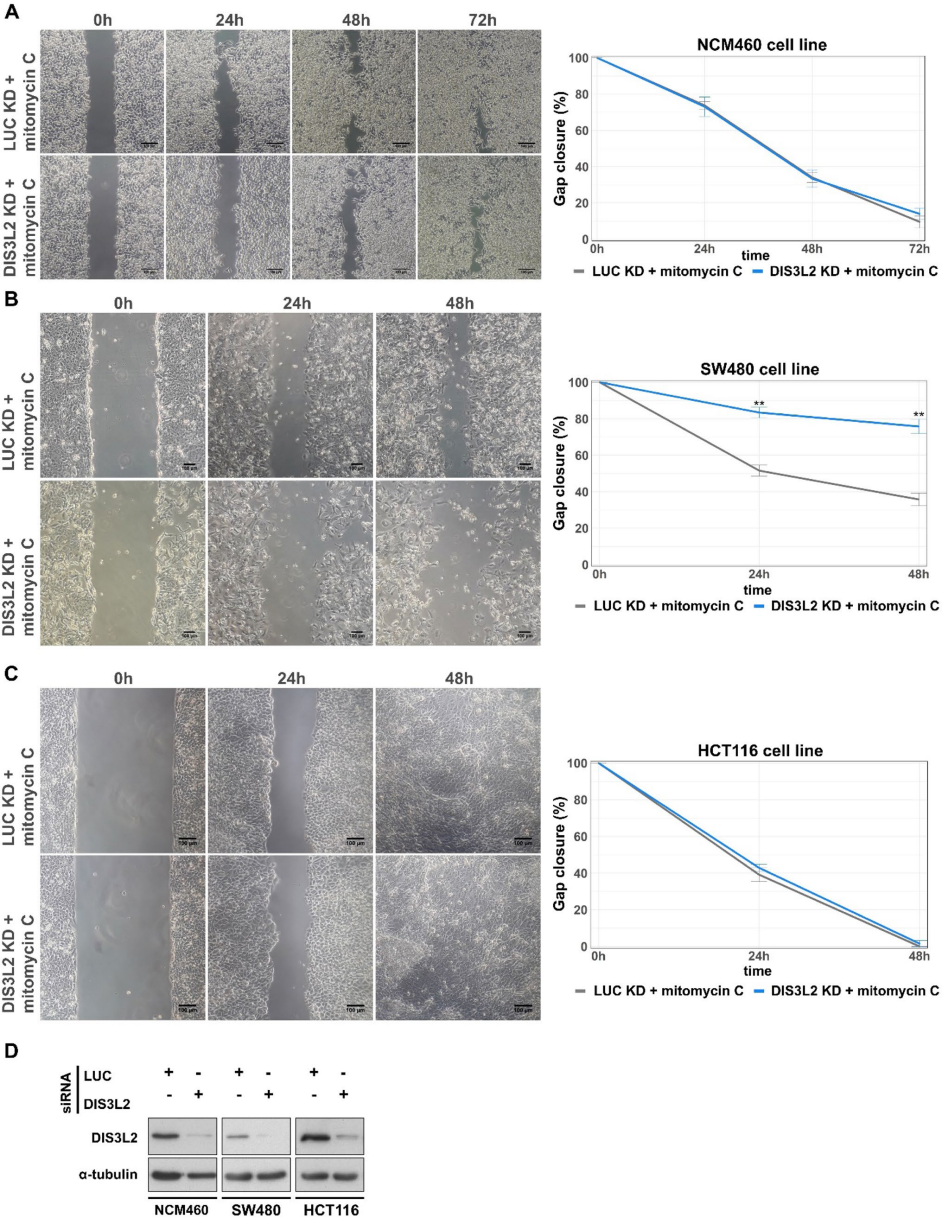
Migration of SW480 cells is impaired by DIS3L2 depletion. Representative images of wound-healing assays conducted in normal colonic NCM460 cells (**A**) and colorectal cancer cell lines SW480 (**B**) and HCT116 (**C**), after mock (siLUC) or DIS3L2 depletion (siDIS3L2). Line plots represent gap closure (%) after scratching at the indicated time points. **D** Representative Western blot analysis for DIS3L2 and α–tubulin (loading control) to monitor DIS3L2 KD efficiencies in each cell line. n = 4, statistical significance relative to mock condition are indicated as: (*) p < 0.05, (**) p < 0.01, and (***) p < 0.001

The above described control (siLUC) and ribonuclease (siDIS3L2) silencing approaches were again applied prior to wounding the cell monolayers and gap closure was monitored for up to 72 h. DIS3L2 depletion did not significantly affect the ability of non-transformed NCM460 cell monolayers to close the wound (Fig. 7A). Well-differentiated Caco-2 and HT-29 CRC cells also showed no significant variation in wound closure (Supplementary Fig. 6A and 6B, respectively). In contrast, DIS3L2 depletion strongly inhibited gap closure of the more mesenchymal-like SW480 cells (Fig. 7B). The effect was already significant at 24 h and reached a 50% difference 48 h after wounding. Interestingly, the also poorly differentiated HTC116 cells did not show a significant difference in the rate of wound closure upon DIS3L2 KD (Fig. 7C). However, it is worth noticing that, in contrast to SW480 cells, HTC116 cells showed a rather erratic behavior, migrating towards and away from the gap, even in mock siLUC conditions. This could be masking changes in the overall migratory behavior of these cells in this assay. Nonetheless, these results indicate that DIS3L2 depletion impairs motility in SW480 cells.

Next we made the reverse experiment, overexpressing human wild type DIS3L2 as above. In line with the MTT cell viability assays, no significant differences in wound closure were observed for any of the 5 cell lines, comparing mock transfected (pcDNA3.1 +) or DIS3L2-overexpressing cells (Supplementary Figs. 8, 9).

### DIS3L2 KD impairs transwell migration and invasion of CRC cells

The somewhat inconclusive results of HTC116 in the wound-closure assay prompt us to use another approach to analyze the impact of DIS3L2 KD in CRC cell migration. Another widely used method assessing cell motility is the transwell assay, also known as Boyden chamber [48, 49], which explores the cells’ chemotactic behavior [50]. Cells were transfected with siLUC (control conditions) or siDIS3L2 and seeded on the upper compartment of the chamber. Cells were then allowed to migrate through the pores of the chamber’s PET membrane into the lower compartment, driven by a gradient of fetal 1% bovine serum (FBS) in the upper compartment and 20% in the lower compartment.

**Fig. 8:**
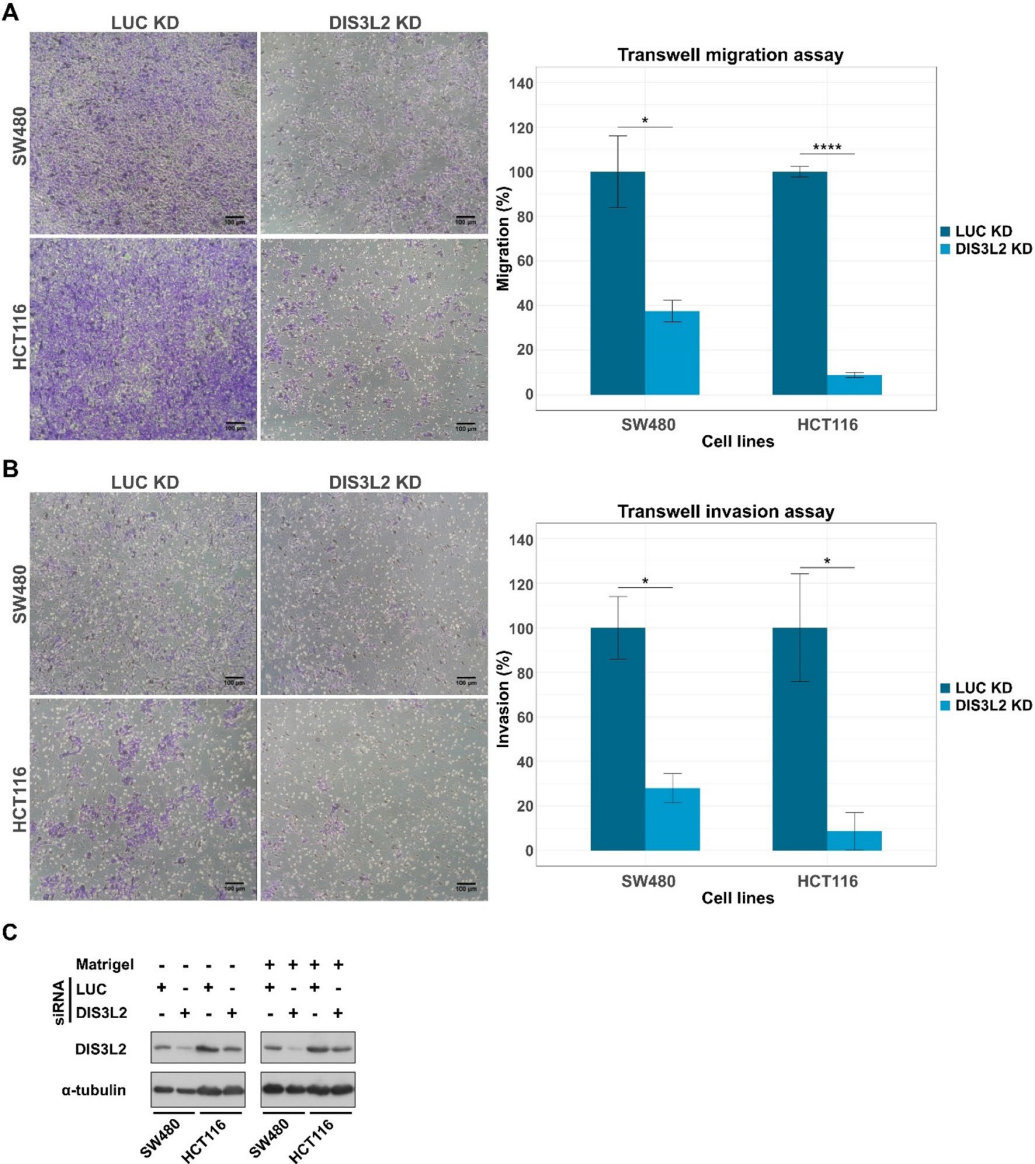
Knockdown of DIS3L2 reduces chemotactic migration and invasion of colorectal cancer SW480 and HCT116 cells. **A** Representative images of transwell migration assays performed in SW480 (top) and HCT116 (bottom) cell lines after mock (siLUC) or DIS3L2 depletion (siDIS3L2). Bar plots represent the quantification of the number of cells migrating to the lower compartment, expressed in % of control condition. **B** Representative images of transwell invasion assays performed in Matrigel-coated chambers. SW480 (top) and HCT116 (bottom) cells were treated as in (**A**). Bar plots represent the quantification of the number of cells invading through the matrix to the lower compartment, expressed in % of control condition. **C** Representative Western blot analysis for DIS3L2 and α–tubulin (loading control) to monitor DIS3L2 KD efficiencies in each cell line. n = 3, statistical significance relative to mock condition are indicated as: (*) p < 0.05, (**) p < 0.01, and (***) p < 0.001

In this assay, after depletion of DIS3L2, the number of HCT116 cells migrating to the lower compartment decreased by over 90%, compared to control conditions (Fig. 8A). Moreover, in agreement with the wound-healing results, the number of migrating SW480 cells was also significantly lower upon DIS3L2 KD, decreasing by more than 64% when compared to siLUC-treated cells (Fig. 8A). NCM460, HT-29 and Caco-2 cells were also subjected to transwell migration assay, but none exhibited detectable variations in migration towards the lower transwell compartment between control and DIS3L2-depleted conditions (Supplementary Fig. 10).

Next, we investigated whether the migration impairment induced by DIS3L2 depletion in SW480 and HTC116 cells would translate to their invasive properties. For this, we repeated the transwell experiments covering the upper compartment membrane with a layer of extracellular matrix-mimicking matrigel. Results showed that, compared to control conditions (siLUC), DIS3L2 KD also induced a significant decrease in the number of SW480 (72%) and HCT116 (93%) cells invading the lower chamber (Fig. 8B), indicating that depletion of DIS3L2 expression can disrupt the migratory and invasive properties of poorly differentiated CRC cells.

## Discussion

The function of DIS3L2 as an exoribonuclease involved in the RNA surveillance pathway of different types of RNAs and its preference by uridylated transcripts has been widely investigated during the last decade [5, 7–10, 37]. However, the role of DIS3L2 in cancer is still poorly understood, namely whether it acts as a driver or suppressor of tumorigenesis [1, 26]. In this regard, our study characterized DIS3L2 as an important promotor of CRC viability and invasive behavior. Our preliminary TCGA data analysis revealed that DIS3L2 mRNA levels are higher in CRC tissues versus non-tumorous tissues. In addition, we observed that high DIS3L2 expression was associated with worse prognosis in patients with advanced CRC (stages III and IV). Similar outcomes have been reported in patients with hepatocellular carcinoma, the major type of primary liver cancer [51]. In this cancer subtype, DIS3L2 mRNA and protein expression were upregulated in cancerous tissues versus matched adjacent noncancerous liver samples [26]. Intriguingly, in the same study it was observed by immunohistochemistry that expression of this ribonuclease increased gradually from TNM stage I–IV. This correlation observed in liver cancer together with the prognostic value of DIS3L2 in the advance stages of CRC suggest a potential role of this ribonuclease in tumor progression. This is in agreement with our findings that depletion of DIS3L2 had little to no effect in the migratory behavior of well-differentiated HT-29 and Caco-2 cells, as well as of non-transformed NCM460 colonocytes, but nearly abolished the invasive abilities of poorly differentiated HCT116 and SW480 CRC cells.

Indeed, using RNA-seq we discovered that DIS3L2 has a broad impact on the transcriptome of SW480 cells. The majority of affected transcripts after DIS3L2 KD are upregulated (Fig. 3A), an expected phenotype given its ribonuclease function. However, a considerable number of transcripts is significantly downregulated (314 transcripts displaying > twofold decrease), most likely representing indirect effects. These results are in line with a previous RNA-seq experiment enriched for mRNAs isolated from DIS3L2-depleted HeLa cells, showing a large disruption of cellular RNA homeostasis [6]. Analysis of common substrates between that set of DIS3L2 mRNA targets in HeLa cells, and the pool of DIS3L2 upregulated mRNAs here detected, revealed a no significant overlap between both gene sets. This robust difference between DIS3L2-targeted mRNAs in HeLa and SW480 cell lines suggests a DIS3L2 substrate recognition in a cell type-specific manner. Due to the vast literature describing the joint action of DIS3L2 with TUTs 4/7, we also included simultaneous downregulation of these three factors in our RNA-seq experiment. Interestingly, the triple KD condition attenuates the global transcriptome changes observed upon the single depletion of DIS3L2. This compensatory effect is in agreement with published data from our lab showing that DIS3L2 + TUTs 4/7 triple KD abolishes the accumulation of a set of DIS3L2/NMD targets that in turn displays increased mRNA levels upon DIS3L2 KD [9]. Both, single and triple depletions predominantly accumulate upregulated mRNAs encoding proteins involved in membrane-trafficking related processes and in a lesser extent, proteins involved in cell cycle regulation. Enriched GO terms in this study are consistent with previous functional annotation clustering reported in DIS3L2-depleted HeLa cells, also showing significant enrichment of upregulated genes in endoplasmic reticulum and Golgi-mediated transport, as well as cell cycle regulation [6]. Analysis of significant downregulated transcripts showed enrichment in multiple GO terms involved in protein synthesis and ribosome biogenesis. Ribosome biogenesis is precisely coordinated along the cell cycle [52] and its activation rate is typically associated with cell proliferation [53]. Moreover, inhibition of rRNA synthesis halts epithelial-to-mesenchymal transition (EMT) and attenuates pro-invasive programs [54]. Thus, the observed enrichment in downregulated mRNAs encoding factors of the ribosome biogenesis machinery is consistent with the impairment of CRC cell migration and invasion observed here upon DIS2L2 silencing. In addition, KEGG pathway enrichment from the pool of DIS3L2 KD downregulated transcripts displayed associations with various cancer and metastasis-associated pathways, which may reflect an oncogenic deceleration. Regarding KEGG term analysis for upregulated mRNAs from both KD conditions, we observed a particular enrichment in association with human neurodegenerative diseases. This is not unexpected, considering the critical role of membrane-trafficking in neuron physiology.

The above transcriptomic outputs prompted us to study the impact of DIS3L2 in cell viability and its potential role in cell migration and invasion. The four CRC cell lines chosen for the present study differ in their oncogenic background and tumorigenic properties. HCT116 is known to be a poorly differentiated and highly invasive cell line, whereas SW480 cells have high metastatic potential but a somewhat less dedifferentiated phenotype than HCT116 cells [41, 55, 56]. On the other hand, Caco-2 and HT-29 cells are better differentiated cells with a low invasive potential [42, 56].

Our data showed that DIS3L2 downregulation significantly reduced the viability of the most dedifferentiated CRC cell lines, SW480 and HCT116, but had little to no effect on the more differentiated Caco-2, HT-29 and non-transformed NCM460 cells. These findings indicate that, as with the migratory and invasive properties, interference with DIS3L2 abundance selectively impact viability of highly oncogenic CRC cells. Consistently, similar results were found in invasive human hepatocellular carcinoma cells, in which silencing of DIS3L2 by siRNA treatment inhibited cell proliferation [26]. In contrast, DIS3L2 silencing in the HEK293 cell line has been reported to promote cell proliferation [14], which supports its cell type-specific function. Remarkably, our data show that triple DIS3L2 + TUTs 4/7 KD recovers, at least partially, cell viability in SW480 and HCT116 CRC cells. This phenomenon is in line with the lower global transcriptomic changes exhibited after the triple KD condition compared with the single KD of DIS3L2 in the RNA-seq experiment, where we identified more similarities in the gene expression profile between control and triple KD conditions than between the triple and single KD conditions. This indicates that cellular effects of DIS3L2 KD are uridylation-dependent, suggesting that uridylation could be, for instance, protecting DIS3L2-targeted transcripts from other degradative pathways. This issue will require further investigation. In contrast, overexpression of DIS3L2 did not affect cell viability in any of the five cell lines here tested. We hypothesized that perhaps more uridylation was needed over DIS3L2 substrates in order to impact cell viability, but the simultaneous overexpression of DIS3L2, TUT4 and TUT7 had no detectable effect in SW480 and HCT116 cells. This suggests that either the DIS3L2-associated pro-viability mechanisms were already fully stimulated in these cells or additional indirect mechanisms are limiting the pro-viability cues from DIS3L2 overexpression. Further investigation is also needed to clarify this matter. For instance, recruitment of TUTs 4/7 at the mRNA 3’ termini previous to DIS3L2 degradation could be upstream regulated by other trans-acting factors, as described for the LIN28-TUTs-DIS3L2 pathway [8, 57–59].

KEGG analysis of the pool of upregulated transcripts upon DIS3L2 depletion displayed a clear enrichment in the mTOR signaling pathway components. The mTOR pathway is a known modulator of key cell cycle regulators, such as cyclin D, which makes this pathway crucial for cell viability and proliferation [43, 60, 61]. Given the inhibitory effect of DIS3L2 KD in cell viability, we explored a potential target of DIS3L2 among genes that clustered in the mTOR pathway, and we found AZGP1, whose mTOR inhibitory activity has been previously documented in different cancer types, including CRC [44–46]. Yu and colleagues showed that overexpression of AZGP1 impairs cell proliferation, migration and invasion via suppression of the mTOR pathway, by decreasing mTOR phosphorylation at Ser2448 which decreases the abundance and activity of several of its downstream targets [44]. These findings are in line with our results, where DIS3L2 depletion leads to upregulation of AZGP1, decreased mTOR and 4EBP phosphorylation and downregulation of cyclin D1 levels in SW480 cells. Moreover, we observed that phosphorylation status of mTOR and 4EBP was restored after DIS3L2 overexpression in siDIS3L2-treated SW480 cells. In addition, we detected a significant positive correlation between *CCND1* and *DIS3L2*, according to transcriptomic data from the TCGA database, providing further evidence for the role of DIS3L2 in the mTOR signaling pathway. Further research is now needed to fully characterize the role of DIS3L2 in cell proliferation, namely via AZGP1-mediated mTOR inhibition.

Cell migration is a key phenomenon during cancer metastasis, since cell motility is a crucial rate-limiting step in tissue invasion by cancer cells from the primary tumor to local and distant sites [62–64]. Our results show that lower DIS3L2 expression impaired migration abilities of SW480 cells, whereas DIS3L2 overexpression did not induce any significant change, in line with results from MTT assays. Chemotaxis choreographs tumor cells behavior, becoming a key factor for tumor dissemination during cancer progression and metastasis [64–66]. Implementation of the transwell assay as a method to test chemotactic cell motility, revealed that depletion of DIS3L2 besides impairing migration in SW480, also impacts motility of HCT116 cells. In addition, matrigel invasion assays showed that knockdown of DIS3L2 significantly reduced the invasion ability of SW480 and HCT116 cells, but not that of NCM460, Caco-2 and HT-29 cell lines, an expected result due to their well-known low invasion abilities [39, 56]. These results indicate that DIS3L2 is required to maintain an invasive phenotype in CRC cells with higher degree of dedifferentiation. Since AZGP1 is also known to have an inhibitory effect towards cell migration [44], the mTOR pathway may also be involved in this behavioral change. DIS3L2 indirect targets, however, may also participate. For instance, we found the expression of *IGF2*, which is known to be influenced by DIS3L2 [15], significantly downregulated in SW480 cells after DIS3L2 KD (-0.81 log2FC). IGF2 is a crucial factor for cancer development and progression and promotes cell migration and invasion [67, 68], so its downregulation could contribute to the observed phenotypes in invasive CRC cells. However, this aspect will require further investigation.

In summary, this study demonstrates that DIS3L2 is overexpressed in colorectal cancer tissues and higher gene expression is associated with more advanced disease and worse patient prognosis. Knockdown of DIS3L2 has a significant impact on the transcriptome of poorly differentiated CRC cells reducing their viability and inhibiting their migratory and invasive properties. Although other studies had described a role for DIS3L2 in sustaining cell viability in different cancer types, to our knowledge, this study is the first to demonstrate its critical role in enabling the invasive behavior of highly dedifferentiated CRC cells. Altogether, these findings expand the knowledge on DIS3L2 role in cancer and suggests that, given its nearly absent effects in well-differentiated and non-transformed cells, the targeting of DIS3L2 may present a novel therapeutic venue to explore in advanced CRC.

### Supplementary Information

Below is the link to the electronic supplementary material.

Supplementary file1 (DOCX 7182 KB)

Supplementary file2 (XLSX 3039 KB)

Supplementary file3 (XLSX 320 KB)

## Data Availability

The datasets generated during the current study are available in the European Nucleotide Archive repository, through study accession number PRJEB59356.
