# Supplementary material for: DIS3L2 knockdown impairs key oncogenic properties of colorectal cancer cells via the mTOR signaling pathway
Source: Cell Mol Life Sci. 2023 Jun 20;80(7):185. doi: 10.1007/s00018-023-04833-5 (PMC10282049; doi:10.1007/s00018-023-04833-5)
Supplement: Supplementary file 1 — Supplementary file1 (DOCX 7182 KB) [file 18_2023_4833_MOESM1_ESM.docx]

DIS3L2 is involved in colorectal cancer via the mTOR pathway by regulating AZGP1 mRNA levels

Juan F. García-Moreno^1,2^ • Rafaela Lacerda^1,2^ • Paulo J. da Costa^1,2^ • Marcelo Pereira^2^ • Margarida Gama-Carvalho^2^ • Paulo Matos^1,2, ¥^ • Luísa Romão^1,2, ¥,*^

^1^Departamento de Genética Humana, Instituto Nacional de Saúde Doutor Ricardo Jorge, 1649-016 Lisboa, Portugal

^2^Faculdade de Ciências, BioISI – Instituo de Biossistemas e Ciências Integrativas, Universidade de Lisboa, 1749-016 Lisboa, Portugal

Supplementary Material

**Supplementary Table 1.** Primer sequences used for RT-qPCR analysis

| **Gene** | **Orientation** | **Sequence (5’- 3’)** |
| --- | --- | --- |
| GAPDH | Forward | CCATGAGAAGTATGACAACAGCC |
| GAPDH | Reverse | GGGTGCTAAGCAGTTGGTG |
| DIS3L2 | Forward | ACCGCGAGAGCAACAAGCT |
| DIS3L2 | Reverse | GATCTTGTGGGCCACTGC |
| TUT4 | Forward | AAAAGGGACCCAGTTTACTGTTG |
| TUT4 | Reverse | GTCCGATACGTCTTCAATTCCTG |
| TUT7 | Forward | ATAACACCAGGGAACTATGGGA |
| TUT7 | Reverse | CATTCATCCAAGCGGGTTGAC |
| CDKN1A | Forward | AGGTGGACCTGGAGACTCTCAG |
| CDKN1A | Reverse | TCCTCTTGGAGAAGATCAGCCG |
| CCND2 | Forward | GGACATCCAACCCTACATGC |
| CCND2 | Reverse | CGCACTTCTGTTCCTCACAG |
| SLC17A7 | Forward | GCAAGTACATCGAGGACGCCAT |
| SLC17A7 | Reverse | GCCACGATGATGGCATAGACTG |
| APLP1 | Forward | GTCCAAGAACCTGCCTAAAGCC |
| APLP1 | Reverse | CTGGTCGTTGATAAGGGCGATG |
| QPCT | Forward | CAAACTCAGCCAGGTGGTTCGA |
| QPCT | Reverse | GGTCATCCTGAATCACACCTCC |
| RGS5 | Forward | GAAGCCAGACTCAGTTGGTGAC |
| RGS5 | Reverse | GGAGTTTGTCCAGGGAATCACG |
| NGFR | Forward | CCTCATCCCTGTCTATTGCTCC |
| NGFR | Reverse | GTTGGCTCCTTGCTTGTTCTGC |
| MPZL3 | Forward | AGAAGAGGAGCAGGTCTGGCTA |
| MPZL3 | Reverse | GGACACAAAGCCTCGCCATACA |
| RAB39B | Forward | GAGCCAGGAAAACGCATCAAGC |
| RAB39B | Reverse | GAAGACCACCTACTGAGTTCCTG |
| CEACAM1 | Forward | CACGCCAATAACTCAGTCACTGG |
| CEACAM1 | Reverse | TTGTGGAGCAGGTCAGGTTCAC |
| CDHR5 | Forward | CCTTGTCCACAAGCACTATGGC |
| CDHR5 | Reverse | GAGGAACGCCTGGTTGTCAAAG |
| GATA3 | Forward | CCTACTACGGAAACTCGGTCA |
| GATA3 | Reverse | GGTAGGGATCCATGAAGCAG |
| AZGP1 | Forward | GGAAGCAGGACAGCCAACTT |
| AZGP1 | Reverse | TTATTCTCGATCTCACAACCAAAC |

**Supplementary Table 2.** Read quality filtering and mapping statistics of paired-end mRNA-seq libraries across all replicates.

| **Parameters** | **LUC** | **DIS3L2** | **DIS3L2+TUTs** | **LUC** | **DIS3L2** | **DIS3L2+TUTs** | **LUC** | **DIS3L2** | **DIS3L2+TUTs** |
| --- | --- | --- | --- | --- | --- | --- | --- | --- | --- |
| Replicate | 1 | 1 | 1 | 2 | 2 | 2 | 3 | 3 | 3 |
| Number of input reads (millions) | 21.09 | 21.21 | 22.77 | 22.85 | 22.44 | 22.13 | 26.37 | 21.10 | 22.96 |
| Average input read length | 282 | 282 | 282 | 282 | 282 | 282 | 282 | 282 | 282 |
| Uniquely mapped reads number (millions) | 17.66 | 17.70 | 19.21 | 19.29 | 18.91 | 18.62 | 22.68 | 18.04 | 19.23 |
| Uniquely mapped reads % | 83.72 | 83.45 | 84.39 | 84.43 | 84.28 | 84.15 | 85.99 | 85.52 | 83.78 |
| Average mapped length | 280.76 | 280.75 | 280.79 | 280.83 | 280.85 | 280.77 | 280.9 | 280.94 | 280.78 |
| Mismatch rate per base % | 0.21 | 0.21 | 0.21 | 0.20 | 0.21 | 0.21 | 0.21 | 0.21 | 0.22 |
| Deletion average length | 1.91 | 1.86 | 1.96 | 1.91 | 1.92 | 1.97 | 1.89 | 1.92 | 1.92 |
| Deletion rate per base | 0.01 | 0.01 | 0.01 | 0.01 | 0.01 | 0.01 | 0.01 | 0.01 | 0.01 |
| Insertion average length | 1.57 | 1.55 | 1.57 | 1.57 | 1.55 | 1.56 | 1.57 | 1.53 | 1.55 |
| Number of reads mapped to multiple loci (millions) | 10.09 | 10.71 | 11.81 | 11.16 | 13.40 | 12.47 | 13.14 | 11.29 | 13.55 |
| % of reads mapped to multiple loci | 4.78 | 5.05 | 5.19 | 4.88 | 5.97 | 5.64 | 4.98 | 5.35 | 5.90 |
| Number of reads mapped to too many loci | 8207 | 9681 | 10101 | 10140 | 12822 | 10538 | 10243 | 11821 | 12395 |
| % of reads mapped to too many loci | 0.04 | 0.05 | 0.04 | 0.04 | 0.06 | 0.05 | 0.04 | 0.06 | 0.05 |

**Supplementary Table 3.** Cell seeding densities and transfection reagents used in MTT assays.

| **Cell line** | **Seeding (nº cells /well)** | | **Transient transfection** | | |
| --- | --- | --- | --- | --- | --- |
|  | **48h incubation** | **72h incubation** | **Lipofectamine 2000 (µL)** | **siRNA (pmol)** | **Plasmids (ng)** |
| NCM460 | 15000\|20000* | 10000\|15000 | 0.5 | 5 | 38 |
| SW480 | 15000\|20000 | 10000\|15000 | 0.4 | 5 | 38 |
| HCT116 | 10000\|15000 | 5000\|10000 | 0.4 | 5 | 38 |
| Caco-2 | 15000\|20000 | 10000\|15000 | 0.5 | 5 | 38 |
| HT-29 | 20000\|30000 | 15000\|20000 | 0.6 | 6 | 38 |

*Before and after | indicates seeding densities to obtain 30% and 60% cell confluences, respectively

**Supplementary Table 4.** Cell seeding densities and transfection reagents used in wound-healing assays.

| **Cell line** | **Seeding** | **Transient transfection** | | |
| --- | --- | --- | --- | --- |
|  | **nº cells /well** | **Lipofectamine 2000 (µL)** | **siRNA (pmol)** | **Plasmids (ng)** |
| HCT116 | 15000\|20000* | 0.5 | 6 | 42 |
| CAco-2 | 20000\|25000 | 0.6 | 6 | 42 |
| HT-29 | 30000\|45000 | 0.6 | 6 | 42 |

*Before and after | indicates seeding densities to obtain 30% and 70% cell confluences, respectively

**Supplementary Table 5.** DIS3L2 knockdown efficiencies in Figure 2.

| **Figure** | **Cell line** | **DIS3L2 knockdown efficiencies** | |
| --- | --- | --- | --- |
|  |  | **DIS3L2 KD** | **DIS3L2 + TUTs** |
| 2A | SW480 | 0.91 | 0.90 |

**Supplementary Table 6.** DIS3L2 knockdown efficiencies in Figure 5.

| **Figure** | **Cell line** | **Time point** | **DIS3L2 knockdown efficiencies** | |
| --- | --- | --- | --- | --- |
|  |  |  | **DIS3L2 KD** | **DIS3L2 + TUTs KD** |
| 5A | NCM460 | 48 h | 0.84 | 0.92 |
|  |  | 72 h | 0.51 | 0.50 |
| 5B | SW480 | 48 h | 0.78 | 0.82 |
|  |  | 72 h | 0.74 | 0.61 |
| 5C | HCT116 | 48 h | 0.52 | 0.48 |
|  |  | 72 h | 0.77 | 0.52 |
| 5D | CACO-2 | 48 h | 0.84 | 0.84 |
|  |  | 72 h | 0.43 | 0.77 |
| 5E | HT-29 | 48 h | 0.52 | 0.79 |
|  |  | 72 h | 0.77 | 0.95 |

**Supplementary Table 7.** DIS3L2 knockdown efficiencies in Figure 6.

| **Figure** | **Cell line** | **Time point** | **DIS3L2 knockdown efficiencies** | |
| --- | --- | --- | --- | --- |
|  |  |  | **DIS3L2 KD** | **DIS3L2 + TUTs KD** |
| 6B | SW480 | 0 h | 0.87 | - |
|  |  | 1 h | 0.76 | - |
|  |  | 2 h | 0.94 | - |
|  |  | 4 h | 0.91 | - |
| 6C | SW480 | - | 0.99 | - |
| 6D | SW480 | - | 0.78 | 0.62 |
| 6E | SW480 | - | 0.81 | - |

**Supplementary Table 8.** DIS3L2 knockdown efficiencies in Figure 7.

| **Figure** | **Cell line** | **DIS3L2 knockdown efficiencies** |
| --- | --- | --- |
|  |  | **DIS3L2 KD** |
| 7D | NCM460 | 0.87 |
|  | SW480 | 0.97 |
|  | HCT116 | 0.81 |

**Supplementary Table 9.** DIS3L2 knockdown efficiencies in Figure 8.

| **Figure** | **Cell line** | **Matrigel** | **DIS3L2 knockdown efficiencies** |
| --- | --- | --- | --- |
|  |  |  | **DIS3L2 KD** |
| 8D | SW480 | - | 0.44 |
|  |  | + | 0.76 |
|  | HCT116 | - | 0.48 |
|  |  | + | 0.43 |


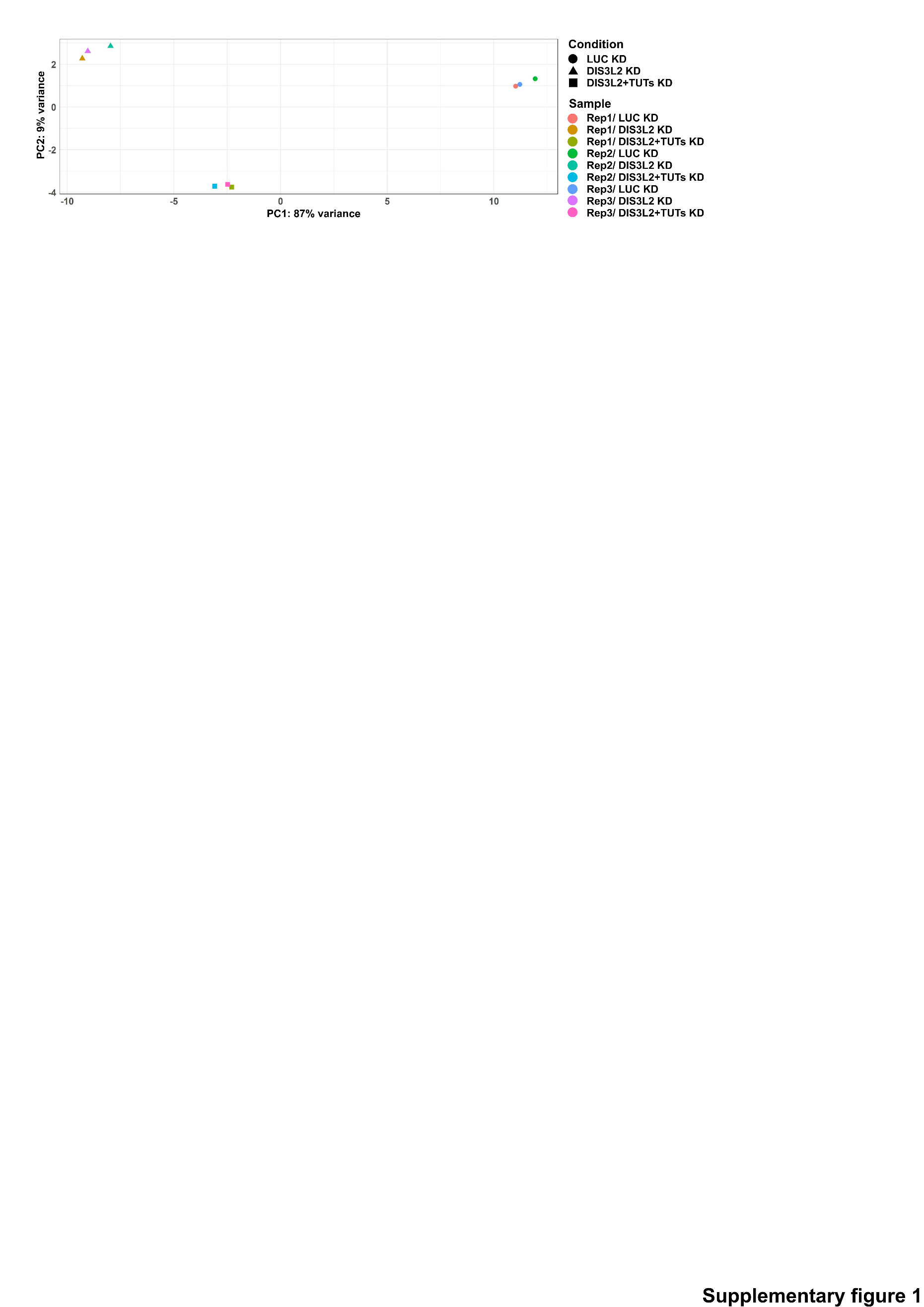


**Supplementary Figure 1.** Principal component (PC) analysis of global expression trends showing the variance among the three knockdown (KD) conditions and replicates. Percentages on each axis indicate amount of variation explained by the principal component 1 (PC1) and 2 (PC2).

**
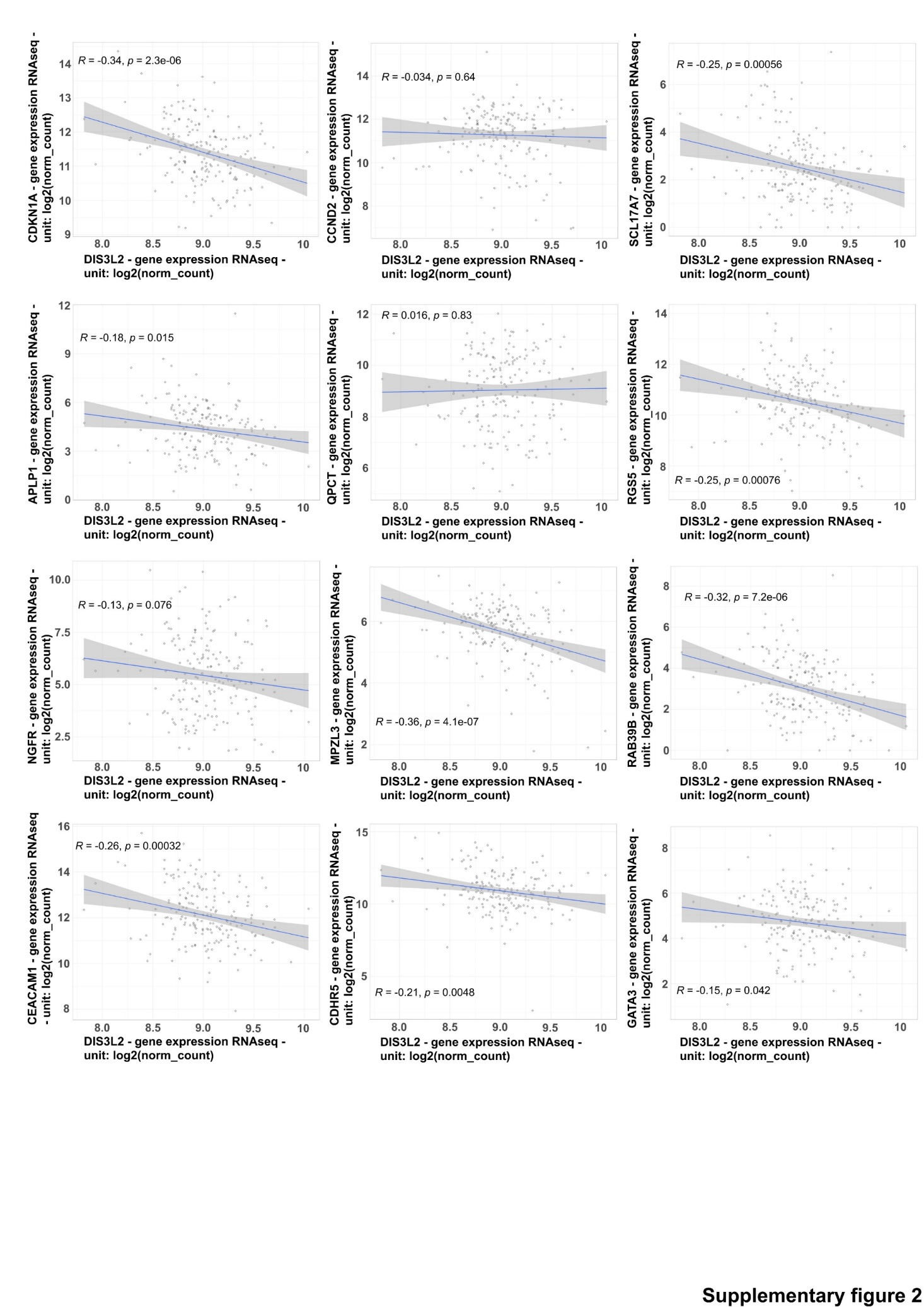
**

**Supplementary Figure 2.** Nine out of 12 genes used for the RNA-seq validation display a significant negative correlation with DIS3L2 mRNA expression levels. Scatter plots represent gene co-expression analyses carried out between DIS3L2 and the set of upregulated transcripts employed in the RNA-seq validation. TCGA RNA-seq data from advanced colorectal cancer samples (stages III and IV) was used to conduct gene co-expression analyses. R indicates Pearson’s correlation coefficient and its associated p-value.

**
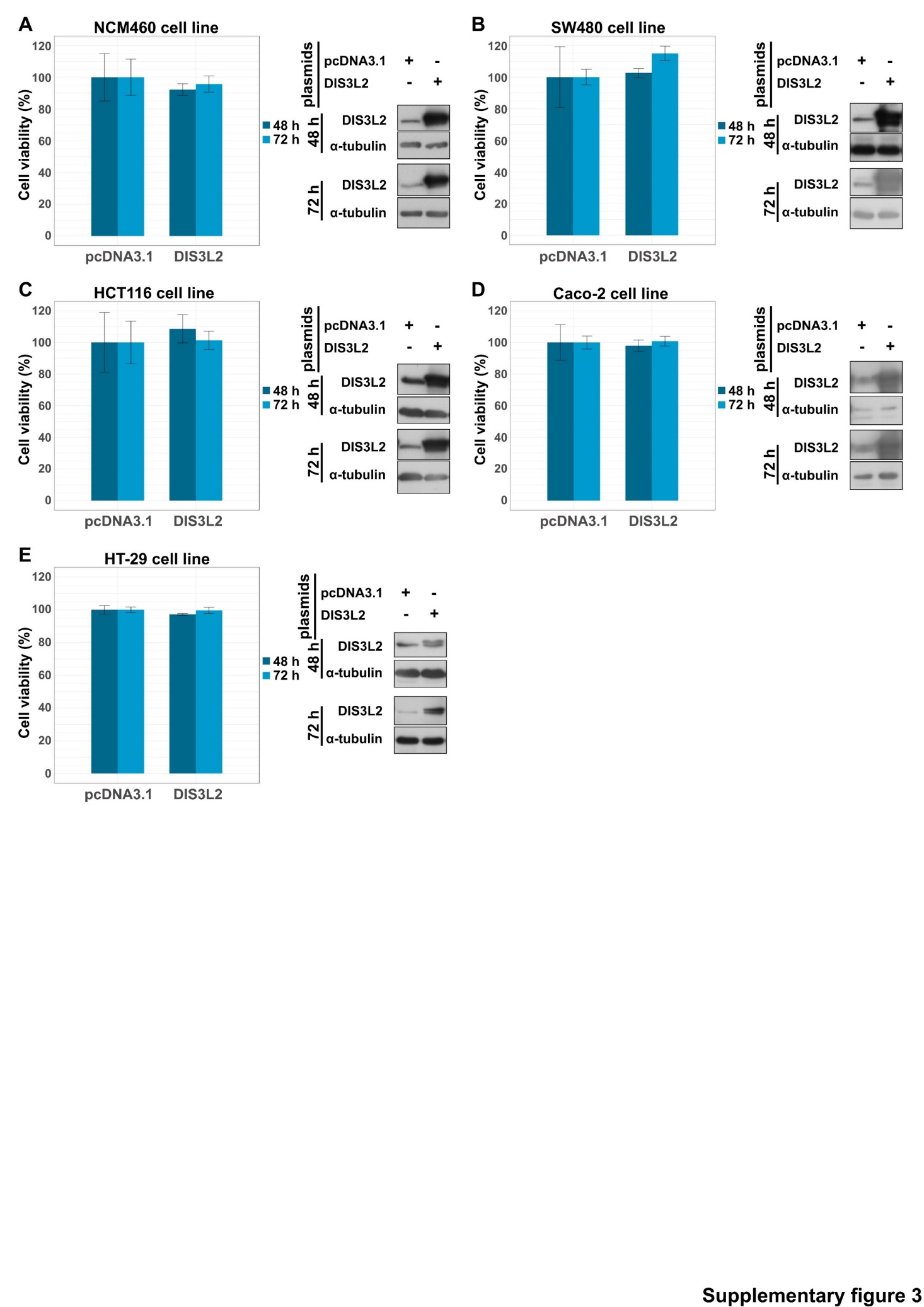
**

**Supplementary Figure 3.** Colon cell viability is not differentially regulated by DIS3L2 overexpression. Histograms represent MTT assays conducted after DIS3L2 overexpression in normal colonic NCM460 cells **(A)** and colorectal cancer cell lines SW480 **(B)**, HCT116 **(C)**, Caco-2 **(D)** and HT-29 **(E)**. MTT assays were performed 48 h and 72 h after post-transfection with the following plasmids: pcDNA3.1 empty vector (control condition) and p3XFLAG-CMV^TM^-10 expression vector encoding wild type DIS3L2. Representative western blots of overexpression efficiencies for each cell line are displayed in the right side of each bar plot. α–tubulin protein was used as internal control in western blot analysis.


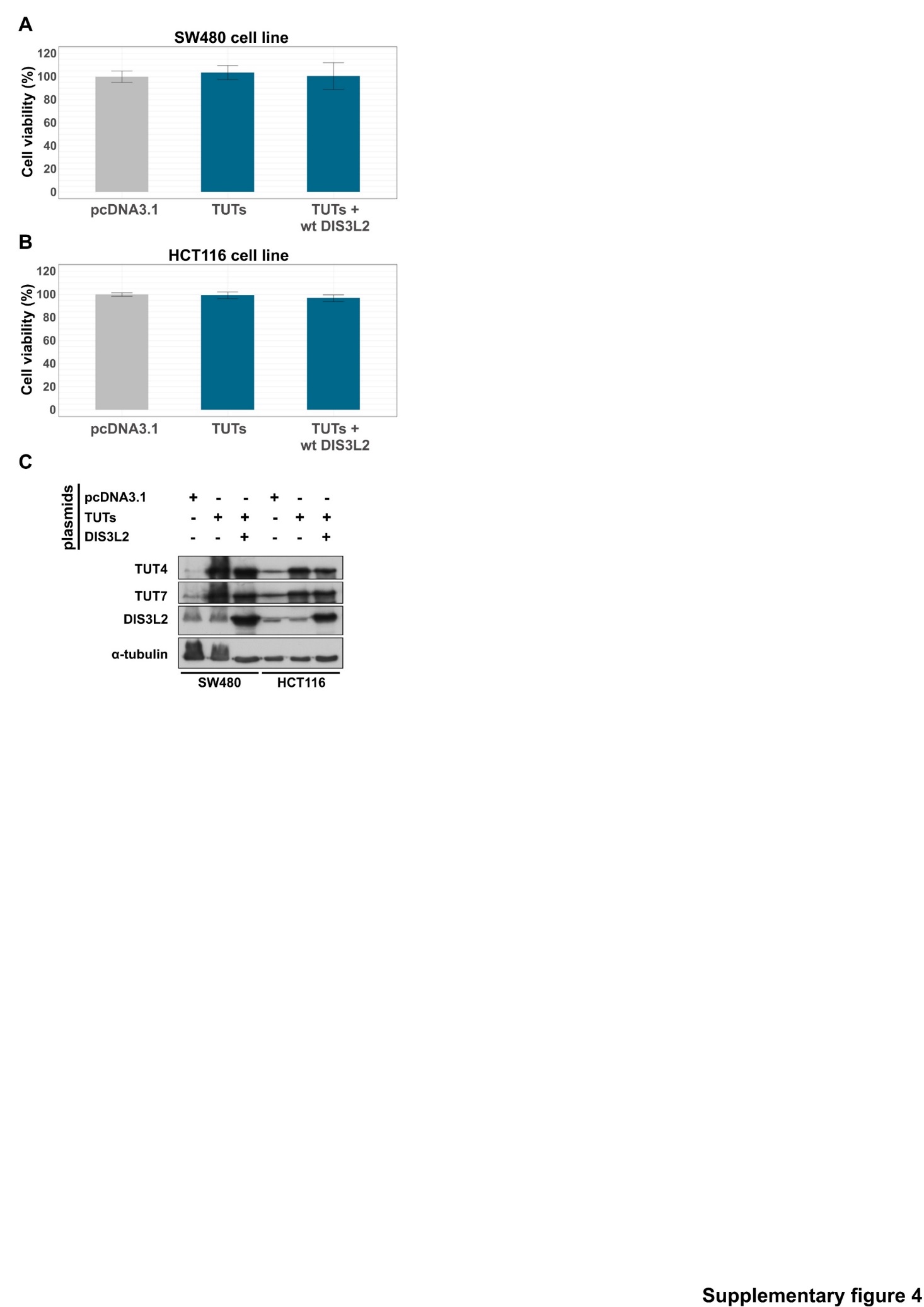


**Supplementary Figure 4.** Overexpression of DIS3L2 and TUTs simultaneously does not interfere with cell viability of colorectal cancer cells. Histograms represent MTT assays conducted after either TUTs or TUTs+DIS3L2 overexpression in SW480 **(A)** and HCT116 cell lines **(B)**. MTT assays were performed 48h after transient transfection of the following plasmids: pcDNA3.1 empty vector (control condition), p3XFLAG-CMV^TM^-10 expression vector encoding either wild type DIS3L2 and pCK-FLAG-TUT4 and pCK-FLAG-TUT7 vectors. **(C)** Representative western blot analysis of DIS3L2 and TUTs overexpression efficiencies for each cell line using α–tubulin protein as internal control.


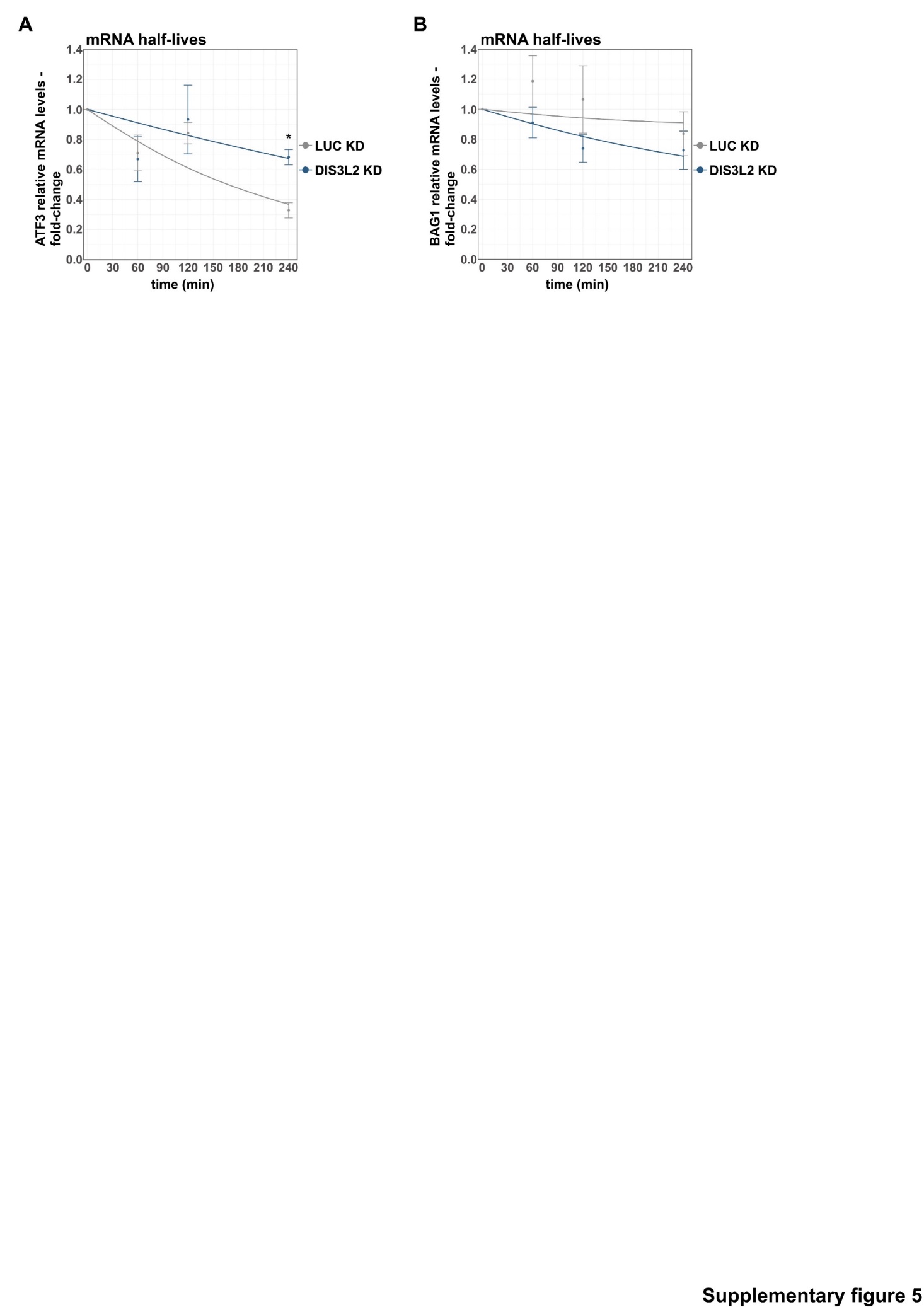


**Supplementary Figure 5.** DIS3L2 is directly involved in the mRNA degradation of ATF3 mRNA in SW480 cells. mRNA stability of ATF3 and BAG1 in SW480 cells transfected with siRNAs targeting LUC (control condition) or DIS3L2. The mRNA levels were determined by RT-qPCR at various time points (0, 60, 120, 240 min) after DRB treatment. ATF3 and BAG1 mRNA decay rates were plotted by normalizing the mRNA level of each time point to that of 0h in each condition; n ≥ 3, statistical significance relative to mock condition are indicated as: (*) p < 0.05.


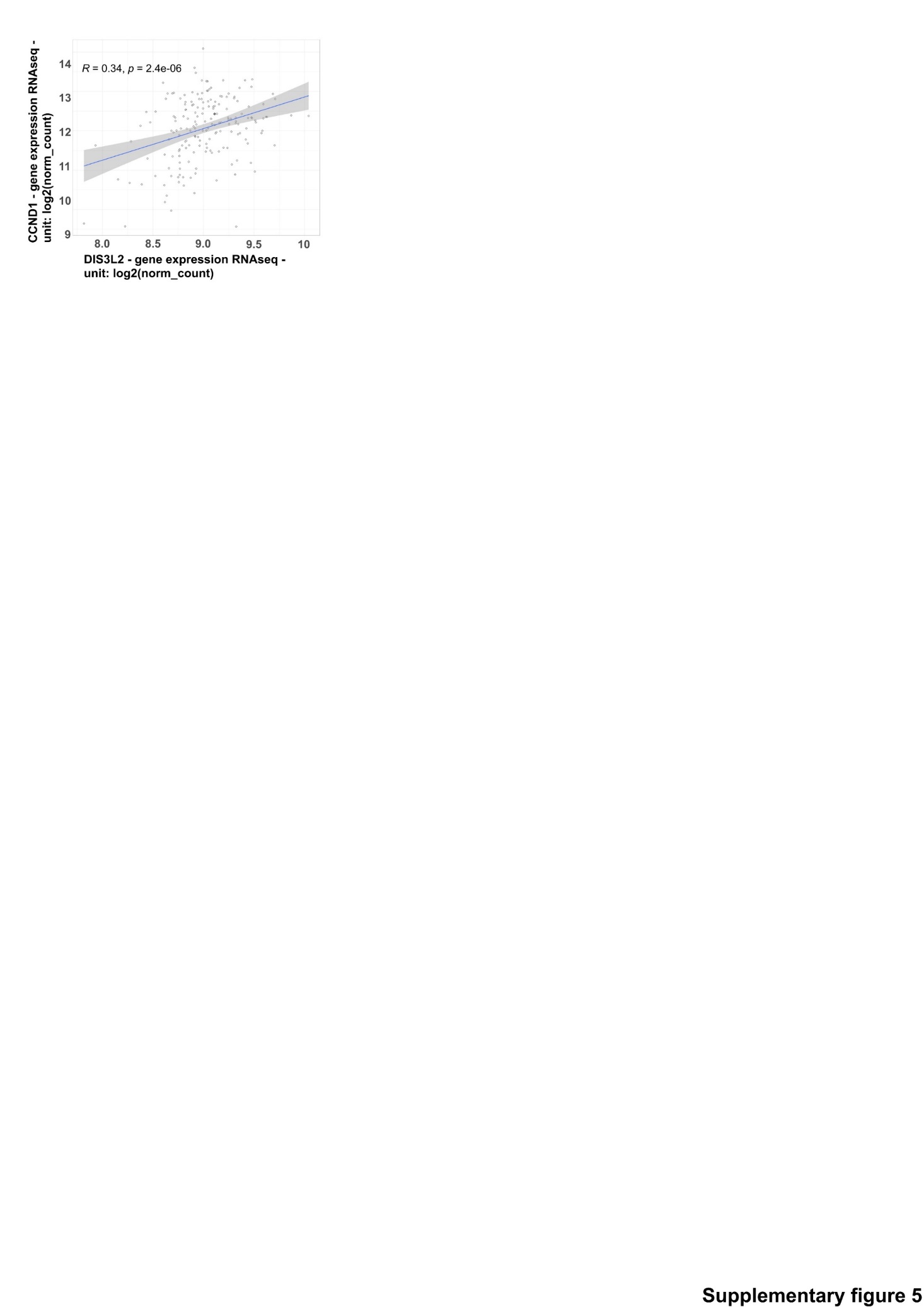


**Supplementary Figure 6.** CCND1 exhibits a significant positive correlation with DIS3L2 mRNA expression levels. Scatter plot illustrates gene co-expression analysis carried out between DIS3L2 and CCND1. TCGA RNA-seq data from advanced colorectal cancer samples (stages III and IV) was used to conduct the gene co-expression analysis. R indicates Pearson’s correlation coefficient and its associated p-value.


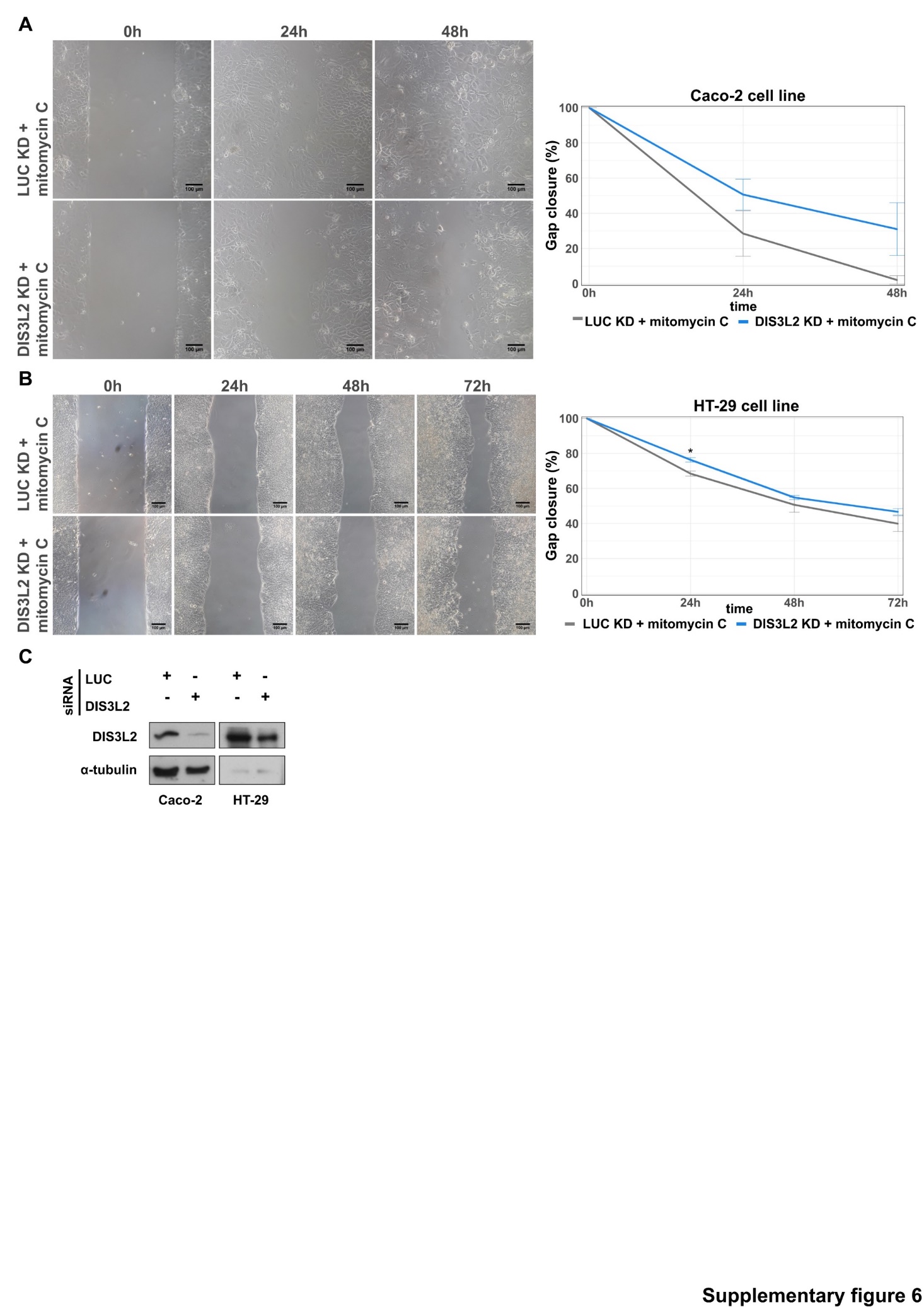


**Supplementary Figure 7.** Knockdown (KD) of DIS3L2 does not induce any significant impact on migration of Caco-2 and HT-29 cell lines. Representative images of wound healing assays performed in colorectal cancer cell lines, Caco-2 **(A)** and HT-29 **(B)**, after mock (LUC KD) or DIS3L2 depletion (DIS3L2 KD). Line plots represent gap closure (%) after scratching at the indicated time points. **(C)** Representative western blot analysis for DIS3L2 and α–tubulin (loading control) to monitor DIS3L2 KD efficiencies in each cell line. Statistical significance relative to mock condition are indicated as: (*) p < 0.05.


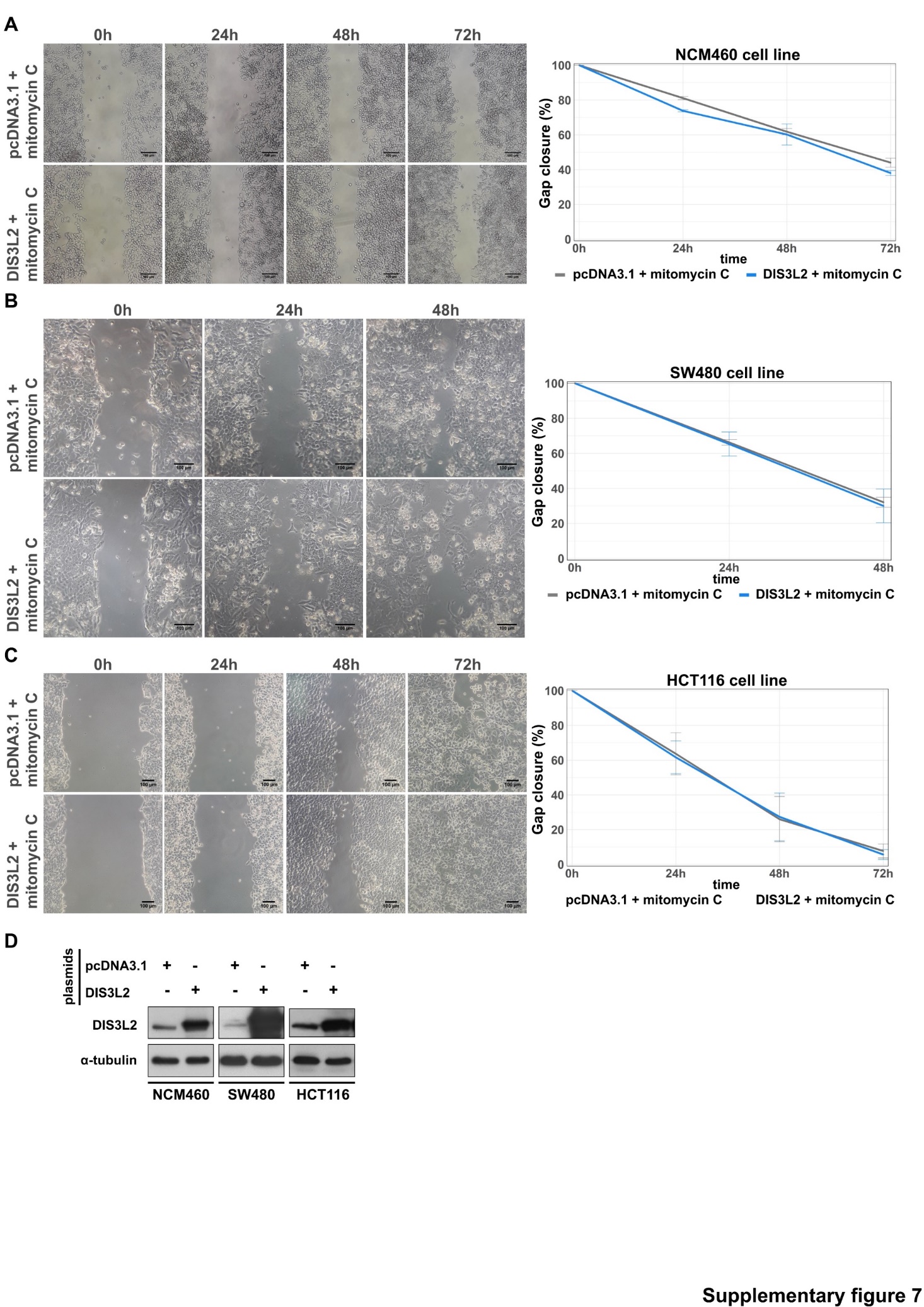


**Supplementary Figure 8.** Colon cell migration is not affected by DIS3L2 overexpression. Representative images of wound healing assays performed in normal colonic NCM460 cells (**A**) and colorectal cancer cell lines SW480 (**B**) and HCT116 (**C**), either mock transfected (pcDNA3.1+; control condition) or overexpressing wild type human DIS3L2. Line plots represent gap closure (%) after scratching at the indicated time points. (**D)** Representative Western blot analysis for DIS3L2 and α-tubulin (loading) to monitor DIS3L2 overexpression efficiencies in each cell line.


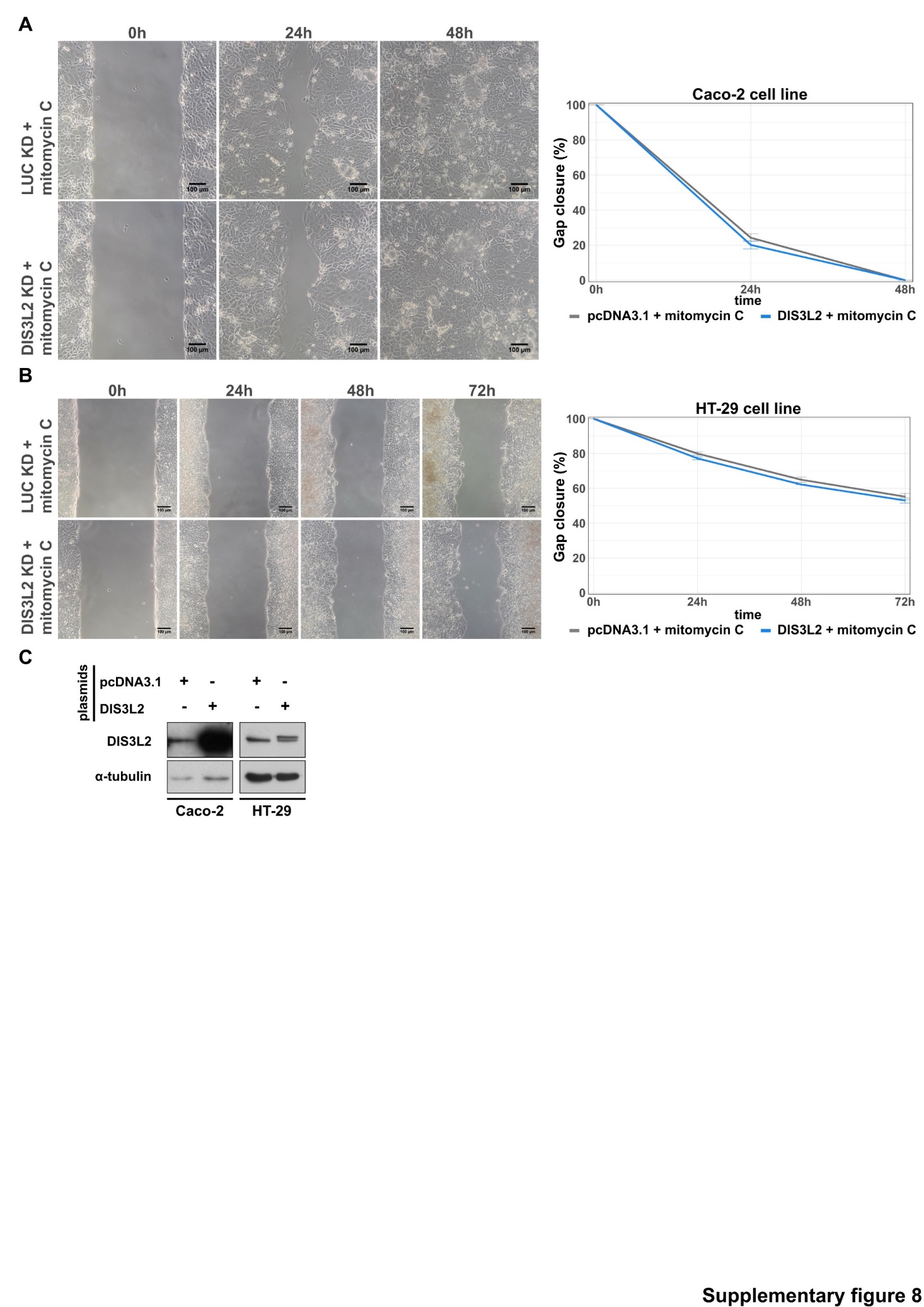


**Supplementary Figure 9.** Overexpression of DIS3L2 does not affect migration of Caco-2 and HT-29 cell lines. Representative images of wound-healing assays performed in colorectal cancer cell lines Caco-2 **(A)** and H-29 **(B)**, either mock transfected (pcDNA3.1+; control condition) or overexpressing wild type human DIS3L2. Line plots represent gap closure (%) after scratching at the indicated time points. **(C)** Representative western blot analysis for DIS3L2 and α–tubulin (loading control) to monitor DIS3L2 overexpression efficiencies in each cell line.


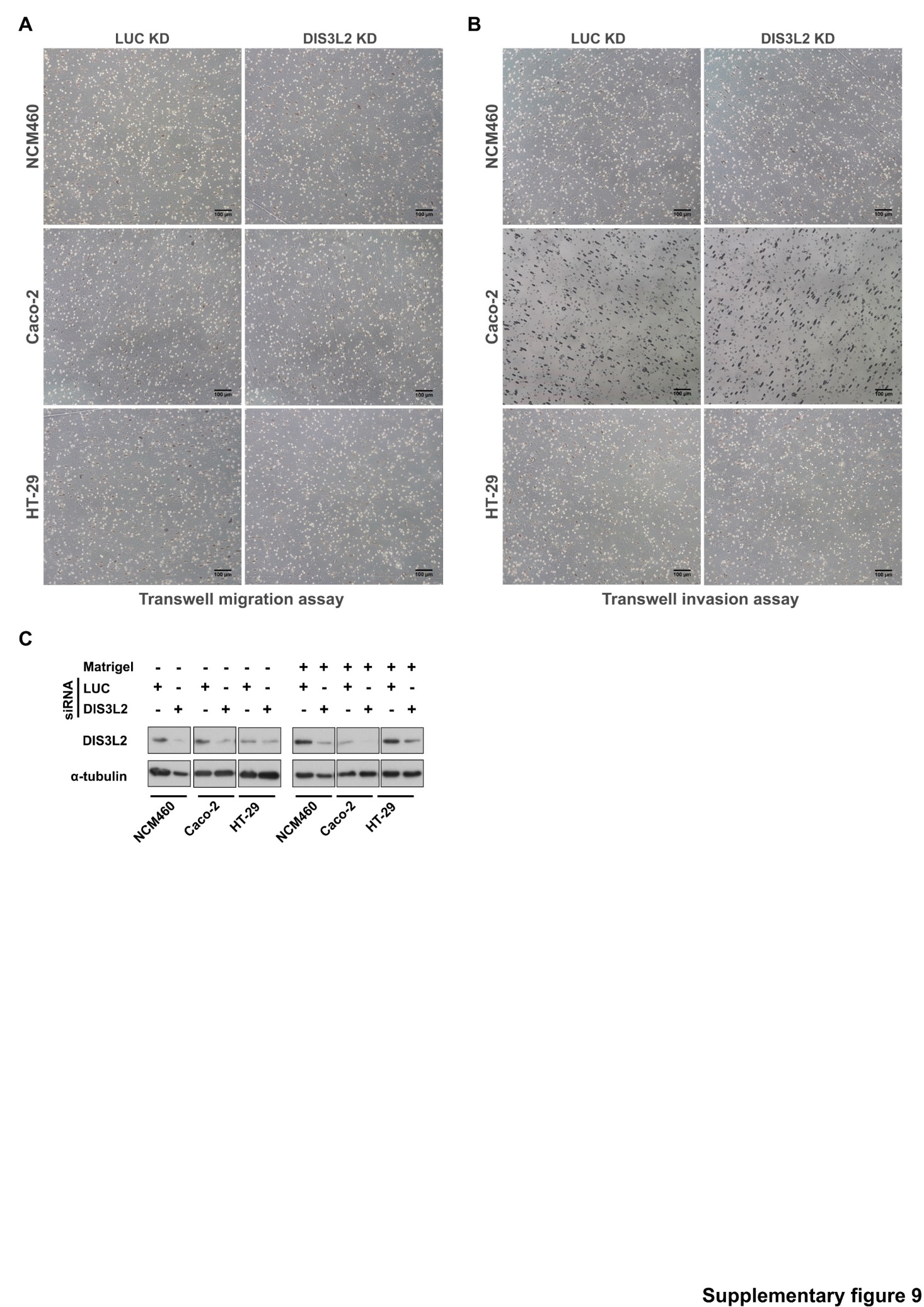


**Supplementary Figure 10.** NCM460, Caco-2 and HT-29 cells does not exhibit the ability to migrate through the transwell membrane. Representative images of transwell migration **(A)** and invasion **(B)** assays performed in NCM460 (top), Caco-2 (middle) and HT-29 (bottom) cells after siRNA treatment targeting either LUC (control condition) or DIS3L2. **(C)** Representative western blot analysis for DIS3L2 and α–tubulin (loading control) to monitor DIS3L2 KD efficiencies in each cell line.
